# Proximity proteomics reveals role of Abelson interactor 1 in the regulation of TAK1/RIPK1 signaling

**DOI:** 10.1002/1878-0261.13374

**Published:** 2023-05-12

**Authors:** Max Petersen, Anna Chorzalska, Makayla Pardo, Anaelena Rodriguez, John Morgan, Nagib Ahsan, Ting C. Zhao, Olin Liang, Leszek Kotula, Paul Bertone, Philip A. Gruppuso, Patrycja M. Dubielecka

**Affiliations:** ^1^ Department of Medicine, Alpert Medical School Brown University Providence RI USA; ^2^ Division of Hematology/Oncology Rhode Island Hospital Providence RI USA; ^3^ Division of Biology and Medicine, Department of Pathology and Laboratory Medicine Brown University Providence RI USA; ^4^ Flow Cytometry and Cell Sorting Core Facility Roger Williams Medical Center Providence RI USA; ^5^ COBRE Center for Cancer Research Development, Proteomics Core Facility Rhode Island Hospital Providence RI USA; ^6^ Department of Chemistry and Biochemistry The University of Oklahoma Norman OK USA; ^7^ Mass Spectrometry, Proteomics and Metabolomics Core Facility, Stephenson Life Sciences Research Center The University of Oklahoma Norman OK USA; ^8^ Department of Surgery Rhode Island Hospital and Warren Alpert Medical School of Brown University Providence RI USA; ^9^ Legorreta Cancer Center, Alpert Medical School Brown University Providence RI USA; ^10^ Department of Urology SUNY Upstate Medical University Syracuse NY USA; ^11^ Department of Biochemistry and Molecular Biology SUNY Upstate Medical University Syracuse NY USA; ^12^ Upstate Cancer Center SUNY Upstate Medical University Syracuse NY USA; ^13^ Division of Pediatric Endocrinology Rhode Island Hospital and Warren Alpert Medical School of Brown University Providence RI USA

**Keywords:** ABL interactor 1, NF‐κB, proximity proteomics, RIPK1, TAK1, TurboID

## Abstract

Dysregulation of the adaptor protein Abelson interactor 1 (ABI1) is linked to malignant transformation. To interrogate the role of ABI1 in cancer development, we mapped the ABI1 interactome using proximity‐dependent labeling (PDL) with biotin followed by mass spectrometry. Using a novel PDL data filtering strategy, considering both peptide spectral matches and peak areas of detected peptides, we identified 212 ABI1 proximal interactors. These included WAVE2 complex components such as CYFIP1, NCKAP1, or WASF1, confirming the known role of ABI1 in the regulation of actin‐polymerization‐dependent processes. We also identified proteins associated with the TAK1‐IKK pathway, including TAK1, TAB2, and RIPK1, denoting a newly identified function of ABI1 in TAK1‐NF‐κB inflammatory signaling. Functional assays using TNFα‐stimulated, *ABI1*‐overexpressing or *ABI1*‐deficient cells showed effects on the TAK1‐NF‐kB pathway‐dependent signaling to RIPK1, with *ABI1*‐knockout cells being less susceptible to TNFα‐induced, RIPK1‐mediated, TAK1‐dependent apoptosis. In sum, our PDL‐based strategy enabled mapping of the ABI1 proximal interactome, thus revealing a previously unknown role of this adaptor protein in TAK1/RIPK1‐based regulation of cell death and survival.

## Graphical abstract

We measured the proximal interactome of an adaptor protein frequently downregulated in cancer—Abelson Interactor 1 (ABI1)—using proximity‐dependent biotin labeling by TurboID followed by mass spectrometry. We identified a mechanism whereby ABI1 regulates TNFR‐mediated NF‐κB‐survival and RIPK1‐apoptotic signaling. ABI1‐deficient cells resisted TNFR‐induced cell death by attenuating RIPK1 S166 phosphorylation, dependent on TAK1 activity and linked to antiapoptotic RIPK1 S321 phosphorylation.

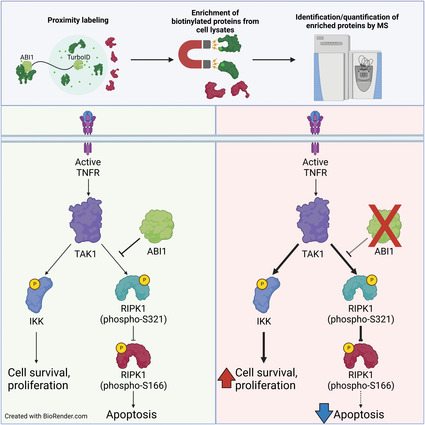

**Abbreviations**
ABI1: Abelson interactor 1
AMP: adenylate monophosphate
BirA: *E. coli* biotin ligase
BP: biological process
c‐ABL: Abelson tyrosine‐protein kinase
c‐CASP3: cleaved caspase 3
c‐FLIPL: CASP8 and FADD like apoptosis regulator
CCCRD: COBRE Center for Cancer Research Development
cIAP: cellular inhibitor of apoptosis protein
CYFIP1: cytoplasmic fragile‐X mental retardation protein 1 (FMR1)‐interacting protein 1
DAB2: disabled homolog 2
DAVID: Database for Annotation, Visualization and Integrated Discovery
DMEM HG: Dulbecco's modified eagle's medium high glucose
EdU: 5‐ethynyl‐2‐deoxyuridine
ERC1: ELKS/RAB6‐interacting/CAST family member 1
FACS: fluorescence‐activated cell sorting
FADD: Fas associated via death domain
Fas: Fas cell surface death receptor
FBS: fetal bovine serum
FC: fold change
FDR: false discovery rate
GAPDH: glyceraldehyde 3‐phosphate dehydrogenase
GFP: green fluorescent protein
GGGGS: glycine–glycine–glycine–glycine‐serine
GO: gene ontology
GSK3B: glycogen synthase kinase‐3 beta
HHR: homeo‐domain homologous region
HPLC: high performace liquid chromatography
HRP: horseradish peroxidase
IgG: immunoglobulin G
IκBα: NF‐κB inhibitor alpha
IKK: inhibitor of nuclear factor kappa‐B kinase
IKKβ: inhibitor of nuclear factor kappa‐B kinase subunit beta
IL‐1: interleukin 1
IRES: internal ribosome entry site
IS: interaction score
KO: knockout
KOC: knockout control
LC–MS/MS: liquid chromatography tandem mass spectrometry
LUBAC: linear ubiquitin assembly complex
MEF: mouse embryonic fibroblast
MPN: myeloproliferative neoplasm
MS: mass spectrometry
MS/MS: tandem mass spectrometry
MSCV: murine stem cell virus retroviral vector system
NCE: normalized collision energy
NCKAP1: non‐catalytic region of tyrosine kinase (NCK) associated protein 1
NEAA: non essential amino acids
NES: nuclear export signal
NF‐κB: nuclear factor kappa‐light‐chain‐enhancer of activated B cells
NP‐40: nonidet P‐40
OD/ID: outer diameter/inner diameter
OE: overexpression
OEC: overexpression control
P/S: penicillin/streptomycin
PA: peak area
PBS: phosphate buffered saline
PDL: proximity dependent labeling
PDL/MS: proximity dependent labeling followed by mass spectrometry
PI3: phosphoinositide 3
PPP6C: serine/threonine‐protein phosphatase 6 catalytic subunit
PSM: peptide spectral match
QQ: quantile‐quantile
RIPA: radioimmunoprecipitation assay buffer
RIPK1: receptor‐interacting serine/threonine‐protein kinase 1
RIPK3: receptor‐interacting serine/threonine‐protein kinase 3
RLR: global robust linear regression normalization
SDS/PAGE: sodium dodecyl‐sulfate polyacrylamide gel electrophoresis
SFK: Src family kinase
SH3: SRC homology 3
SQSTM1: sequestosome 1
SRC: proto‐oncogene tyrosine‐protein kinase Src
STAT3: signal transducer and activator of transcription 3
T‐SNARE: target SNAP receptor
TA: TurboABI1
TAB1: TGF‐beta activated kinase 1/TAK1 binding protein 1
TAB2: TGF‐beta‐activated kinase 1 and MAP3K7‐binding protein 2
TAB3: TGF‐beta activated kinase 1 and TAK1 binding protein 3
TAK1/MAP3K7: transforming growth factor beta‐activated kinase 1/Mitogen‐activated protein kinase kinase kinase 7
TBK1: TANK binding kinase 1
TBS‐T: Tris‐Buffered Saline, 0.1% Tween® 20 Detergent
TC: TurboControl
TMT: tandem mass tag
TNFαa: tumor necrosis factor alpha
TNFR: tumor necrosis factor receptor
TNFR1: tumor necrosis factor receptor 1
TNIP1: TNFAIP3 interacting protein 1
TRADD: tumor necrosis factor receptor type 1‐associated DEATH domain protein
TRAF2/5: TNF receptor‐associated factor 2
TRAF3IP2: TRAF3 interacting protein 2
UI: user interface
VASP: vasodilator‐stimulated phosphoprotein
VSN: variance stabilizing normalization
WASF1: Wiskott‐Aldrich syndrome protein‐family member 1
WAVE: Wiskott‐Aldrich syndrome protein‐family verpolin‐homologous protein
WAVE2: Wiskott‐Aldrich syndrome protein‐family verpolin‐homologous protein 2
WT: wild type
Z‐VAD(‐FMK): carbobenzoxy‐valyl‐alanyl‐aspartyl‐[O‐methyl]‐(fluoromethylketone)

## 1 Introduction

Signal transduction is central to the maintenance of cellular homeostasis, control of cell metabolism, response to external environmental cues, cell growth, proliferation, and death. Signaling dysregulation drives all malignancy. The functional nodes of signal transduction are structured complexes comprising multiple proteins. Resolving the composition, organization, and regulation of these complexes enables new therapeutic approaches that are better targeted to a protein's specific dysregulated context. Adaptor proteins lack enzymatic activity but contain structural motifs that facilitate both protein–protein interactions and context‐specific organization of protein signaling complexes. Adaptor proteins are frequently dysregulated in cancer. Therefore, detailed mapping of adaptor protein interactomes has the potential to offer new insights into cancer pathophysiology and therapy [1].

ABI1 is an adaptor protein containing a C‐terminal SH3 domain, multiple proline‐rich regions, a homeodomain homologous region (HHR), several phosphosites, and an N‐terminal T‐SNARE domain. ABI1 regulates actin cytoskeleton organization through interaction with VASP, WAVE, and associated proteins [2, 3, 4, 5]. ABI1 is a key cell signaling regulatory protein, relaying information from growth factor receptors [6] to PI3 kinase, c‐ABL, or SRC kinases to promote coordinated actin cytoskeleton, cell growth, and proliferation instruction [7, 8, 9]. Both elevated and decreased levels of ABI1 are associated with different types of cancer [10, 11, 12, 13, 14, 15, 16, 17, 18, 19, 20, 21, 22, 23]. Recently, we found evidence of decreased ABI1 expression in hematopoietic stem/progenitor cells in patients with myeloproliferative neoplasm (MPN). We also showed that murine bone marrow‐targeted depletion of ABI1 was associated with an MPN‐like phenotype mechanistically linked to activation of SRC‐family kinases (SFKs), STAT3, and nuclear factor kappa B (NF‐κB) pathways [24]. Considering these findings, we used proximity‐dependent labeling (PDL) to detail mechanistic links between ABI1 and SFKs, STAT3, and NF‐κB and uncovered fundamental insight into the role of this adapter protein in cancer.

The balance between cell death and survival is frequently shifted in cancer. Tumor necrosis factor receptor 1 (TNFR1) signaling is one such pathway that induces either cell survival or death upon activation by TNFα. TNFα stimulation induces formation of membrane‐proximal TNFR complex I, comprising TNFR1, TNFR1‐associated death domain protein (TRADD) [25], TNFR‐associated factor 2/5 (TRAF2/5) [26], cellular inhibitors of apoptosis (cIAPs) [27, 28], linear ubiquitin chain assembly complex (LUBAC) [29], and receptor interacting serine/threonine protein kinase 1 (RIPK1) [30]. TRADD recruits RIPK1 to the TNFR complex I to be polyubiquitinated by TRAF2/5, cIAPs, and LUBAC E3 ubiquitin ligases to form a network of ubiquitin chains that facilitate recruitment of downstream proteins [28, 31, 32]. A protein complex comprising TAK1, TAB1, TAB2, and TAB3 binds to complex I RIPK1 K63‐linked polyubiquitin chains to promote TAK1 Thr184/187 autophosphorylation [33]. Autophosphorylated TAK1 phosphorylates and activates IKKβ [34, 35], which in turn phosphorylates IκBα, resulting in its subsequent degradation and nuclear translocation of NF‐κB to activate prosurvival and proinflammatory gene transcription [36, 37]. Opposing the prosurvival arm of TNFR signaling, deubiquitinated or incompletely ubiquitinated RIPK1 can autophosphorylate itself at S166 to associate with the Fas‐associated death domain (FADD), c‐FLIPL and pro‐Caspase‐8 [28, 38, 39], forming cytosolic complex IIb. Complex IIb promotes caspase‐dependent apoptosis and caspase‐mediated degradation of RIPK1 and RIPK3 [40].

Proximity‐dependent labeling followed by mass spectrometry (PDL/MS) is a powerful approach to identify both directly and indirectly interacting protein partners involved in steady‐state and transient cell signaling. PDL uses cellular expression of a fusion protein comprising a bait and inducible enzyme that catalyzes production of reactive substrates, which tag proximal proteins. Several methods for PDL in living cells are available, including a biotinylation/streptavidin‐affinity capture‐based method enabled by TurboID. TurboID is an *Escherichia coli*‐derived biotin ligase, catalytically enhanced through directed evolution, which can be genetically fused to a protein of interest. Cellular TurboID is induced by exogenous biotin addition to produce reactive biotinoyl‐5'‐AMP, which rapidly binds exposed lysine residues on proximal proteins. Biotinylated proteins are pulled down from cell lysates using streptavidin affinity, then identified by MS to map the bait protein's proximal interactome [41, 42].

Here we report the identification of the ABI1 proximal interactome using TurboID‐based proximity labeling coupled with label‐free, quantitative MS. PDL data are complemented with functional validations, using both ABI1 overexpressing (ABI OE) and ABI1 deficient (ABI KO) cells. Our obtained PDL results, while confirming the known role of ABI1 in the regulation of actin polymerization‐dependent processes, reveal a new function of this adaptor protein in regulation of RIPK1 in TNFR‐mediated cell death signaling.

## 2 Materials and methods

### 2.1 Vectors and cloning

Information and sequences for primers and vectors used are available in Table S1. MSCV‐TurboID‐IRES‐GFP construction: V5‐TurboID‐NES_pCDNA3 was a gift from Alice Ting (Addgene plasmid # 107169; http://n2t.net/addgene:107169; RRID:Addgene_107169 [41]). The TurboID coding sequence was amplified from V5‐TurboID‐NES_pCDNA3 using primers pTurboID_XhoI‐Kozak FOR, and pTurboID_STOP‐EcoRI REV. This amplicon was gel‐extracted, then restriction ligated into MSCV‐IRES‐GFP_PD using XhoI and EcoRI restriction enzymes. The resulting mixture was transformed into Stable3 competent *E. coli* (Thermo Fisher, Waltham, MA, USA; C737303) and plated for single colonies. Colonies were selected by carbenicillin resistance, and a purified MSCV‐TurboID‐IRES‐GFP sequence was confirmed by Sanger sequencing (Table S2). MSCV‐TurboID‐Linker‐ABI1‐IRES‐GFP construction: pcDNA3.1(+)‐N‐TurboID‐13xGGGGS linker‐ABI1 (ENSMUST00000153931.7) was synthesized in collaboration with Creative Biogene (Shirley, NY, USA) and includes 5' XhoI and 3' EcoRI restriction for cloning into MSCV‐IRES‐GFP_PD as above. The resulting vector MSCV‐TurboID‐Linker‐ABI1‐IRES‐GFP sequence was confirmed by Sanger sequencing (Table S3).

### 2.2 Retrovirus production

Using a scaled‐up Lipofectamine 3000 transfection protocol, HEK293T cells were cotransfected with 1:1 MSCV‐TurboID‐IRES‐GFP and EcoPack packaging vector, or 1:1 MSCV‐TurboID‐Linker‐ABI1‐IRES‐GFP and EcoPack, in 10‐cm dishes using 20 μg total DNA per transfection. Supernatant containing MSCV‐TurboID‐IRES‐GFP or MSCV‐TurboID‐Linker‐ABI1‐IRES‐GFP retrovirus was collected 24 and 48 h posttransfection, separated into aliquots, and frozen.

### 2.3 Single cell‐derived cell lines

NIH/3T3 [ATCC (Manassas, VA, USA), RRID:CVCL_0594, authenticated by Short Tandem Repeat (STR) profiling within the last 3 years by ATCC] grown in complete DMEM HG [DMEM High glucose (Gibco, Grand Island, NY, USA, 31053028), 10% FBS (Corning, Corning, NY, USA, 35‐010‐CV), 1× GlutaMAX (Gibco, 35050‐061), 1× sodium pyruvate (Gibco, 11360‐070), 1× MEM NEAA (Gibco, 11140‐050)] + 1× penicillin/streptomycin (P/S; Thermo Fisher, 15070063) to 70% confluency were transduced by two rounds of infection by 5 h incubation with retroviral supernatants containing 5 μg·mL^−1^ polybrene (EMD Millipore, Burlington, MA, USA, TR‐1003‐G) followed by overnight recovery in culture conditions. After infection, cells were checked for GFP expression by flow cytometry and immunofluorescence microscopy. Single‐transduced NIH/3T3 cells were sorted using a Modular Flow (MoFlo) high‐speed cell sorter (Beckman Coulter, Brea, CA, USA) based on GFP (Fig. S1) into individual wells of 96‐well culture plates containing complete DMEM HG + 1× P/S + 1× antibiotic/antimycotic (Thermo Fisher, 15240096) then expanded to establish cell lines. Cell line characterization by GFP expression level was conducted using a BD II LSR flow cytometer (Fig. S1). Generated cell lines are routinely tested for mycoplasma using the MycoAlert mycoplasma Detection Kit (Lonza, Basel, Switzerland, LT07‐318) and DAPI staining. All experiments were performed in mycoplasma‐free cells.

### 2.4 Immunofluorescence imaging

Preparation and staining for immunofluorescence imaging were performed as described previously [2]. Information about antibodies and stains is provided in Table S1. Confocal images were taken on a Nikon C1si (Tokyo, Japan) confocal microscope.

### 2.5 Immunoblotting

SDS/PAGE was performed in 1× NuPAGE MES SDS Running Buffer (Thermo Fisher, NP0002). Gel electrophoresis was run on gradient gels (Thermo Fisher, WG1402BOX). Protein transfer to nitrocellulose membranes was performed in transfer buffer containing 20% methanol and 1× NuPAGE Transfer Buffer (Thermo Fisher, NP00061). Blocking was conducted using 5% milk in TBS‐T, except for streptavidin, BirA (TurboID), and phosphorylated protein blots which used 5% BSA in TBS‐T. Primary and secondary antibody incubations were conducted in 2% milk in TBS‐T (except for streptavidin, BirA, and phosphorylated protein blots, which used 2% BSA in TBS‐T). Signal was detected using SuperSignal West Pico PLUS Chemiluminescent Substrate (Thermo Fisher, 34579). Information about antibodies is provided in Table S1.

### 2.6 Wound healing assay

Stable NIH/3T3 cell lines expressing TurboControl or TurboABI1 (*n* = 6) were seeded in 6‐well culture plates at 1e6 cells per well and incubated overnight at 37 °C, 5% CO_2_. 100% confluent cells were washed with PBS, scratched with a 10 μL pipette tip, washed again, then imaged at timepoints indicated in Fig. S2, until wound closure. Images were captured using ECHO Revolve microscope system (San Diego, CA, USA) at 10× magnification. Wound healing was quantified using imagej (Bethesda, MD, USA) [43], and statistical comparisons were made between TurboControl and TurboABI1 groups using Student's two‐tailed *t*‐test.

### 2.7 EdU incorporation analysis

Cell lines from TurboControl and TurboABI1 (*n* = 6) were plated in 6‐well plates at 2e5 cells per well then incubated overnight at 37 °C, 5% CO_2_. EdU incorporation and analysis were conducted using Click‐iT Plus EdU Flow Cytometry Assay Kit with Alexa Fluor 647 picolyl azide (Thermo Fisher, C10634), according to the manufacturer's protocol. EdU incorporation was measured using a BD II LSR flow cytometer. Statistical comparisons were made between groups using Student's two‐tailed *t*‐test.

### 2.8 Proximity labeling and biotinylated protein enrichment

1.8e7 total cells per cell line were plated onto 10‐cm tissue culture dishes, at 7.5e5 cells per dish containing complete DMEM culture medium and 1× P/S. Cells were incubated at 37 °C, 5% CO_2_ overnight, and labeling was induced by adding biotin (Invitrogen, Waltham, MA, USA, B1595) from fresh 0.2 μm filtered 100 mm stock in DMEM to a final biotin concentration of 50 μm, then mixed by tilting. Plates were incubated at 37 °C, 5% CO_2_ for 3 h before labeling was stopped by aspirating medium and washing twice with ice‐cold PBS. Cells were scraped and pelleted at 300× ***g*** for 10 min while maintaining cold conditions. Cell pellets were lysed in 1 mL RIPA (Thermo Fisher, 89900) containing inhibitors (1 mm sodium orthovandate, 10 mm sodium pyrophosphate, 10 mm sodium fluoride, 1× protease inhibitor cocktail set III (EMD Millipore, 539134)). Lysis was conducted on ice for 15 min with vortexing every 5 min, then lysates cleared by centrifugation at 4 °C for 15 min at 20 000× ***g***. Magnetic streptavidin beads (Pierce, Waltham, MA, USA, 88817) were washed in lysis buffer with inhibitors three times before adding cleared lysates and incubating overnight at 4 °C with rotation. Bound beads were then washed three times with 1 mL RIPA plus inhibitors and resuspended in 0.3 mL RIPA plus inhibitors before transport to the RIH CCCRD proteomics core facility. Small‐scale biotinylation experiments were conducted as above but using 1e6 cells per 10‐cm dish, and 500 μm biotin for 10 min, for analysis by SDS/PAGE.

### 2.9 Label‐free proteomics

Label‐free proteomics was performed as previously described [44]. Samples were exposed to overnight on‐bead tryptic digestion at 37 °C on a rotator. Tryptic peptides were desalted using C18 Sep‐Pak plus cartridges (Waters, Milford, MA, USA) and were lyophilized for 48 h to dryness. The dried peptides were reconstituted in 30 μL of buffer A (0.1 m acetic acid) and 5 μL was injected for each analysis.

The LC–MS/MS was performed on a fully automated proteomic technology platform that includes an Agilent 1200 Series Quaternary HPLC system (Agilent Technologies, Santa Clara, CA, USA) connected to a Q Exactive Plus mass spectrometer (Thermo Fisher Scientific, Waltham, MA, USA). The LC–MS/MS set up was used as described [44]. Briefly, the peptides were separated through a linear reversed‐phase 90 min gradient from 0% to 40% buffer B (0.1 m acetic acid in acetonitrile) at a flow rate of 3 μL·min^−1^ through a 3‐μm × 20 cm C18 column (OD/ID 360/75, Tip 8 μm, New Objectives, Woburn, MA, USA) for a total of 90 min run time. The electrospray voltage of 2.0 kV was applied in a split‐flow configuration, and spectra were collected using a top‐9 data‐dependent method. Survey full‐scan MS spectra (m/z 400–1800) were acquired at a resolution of 70 000 with an AGC target value of 3 × 10^6^ ions or a maximum ion injection time of 200 ms. The peptide fragmentation was performed via higher‐energy collision dissociation with the energy set at 28 normalized collision energy (NCE). The MS/MS spectra were acquired at a resolution of 17 500, with a targeted value of 2 × 10^4^ ions or maximum integration time of 200 ms. The ion selection abundance threshold was set at 8e2 with charge state exclusion of unassigned and *z* = 1, or 6–8 ions and dynamic exclusion time of 30 s.

### 2.10 Database search and label‐free quantitative analysis

Database search and label‐free quantitative analysis was performed as previously described [45]. Peptide spectrum matching of MS/MS spectra of each file was searched against the Uniprot *Mus musculus* database (TaxonID: 10090, downloaded on 02/09/2015) using the Sequest algorithm within proteome discoverer v 2.3 software (Thermo Fisher Scientific, San Jose, CA, USA). The Sequest database search was performed with the following parameters: trypsin enzyme cleavage specificity, two possible missed cleavages, 10 ppm mass tolerance for precursor ions, and 0.02 Da mass tolerance for fragment ions. Search parameters permitted dynamic modification of methionine oxidation (+15.9949 Da) and static modification of carbamidomethylation (+57.0215 Da) on cysteine. Peptide assignments from the database search were filtered down to a 1% FDR (false discovery rate). The relative label‐free quantitative and comparative among the samples were performed using the Minora algorithm and the adjoining bioinformatics tools of the proteome discoverer 2.3 software.

### 2.11 TMT proteomics and data analysis

TMT proteomics and data analyses were performed as previously described [46, 47]. Proteins from flash‐frozen cell pellets (1e6 cells per pellet, *n* = 3 per cell line) were reduced, alkylated, and purified by chloroform/methanol extraction prior to digestion with sequencing grade modified porcine trypsin (Promega, Madison, WI, USA). Tryptic peptides were labeled using tandem mass tag isobaric labeling reagents (Thermo Fisher) following the manufacturer's instructions and combined into one 10‐plex sample group. The labeled peptide multiplex was separated into 46 fractions on a 100 × 1.0 mm Acquity BEH C18 column (Waters) using an UltiMate 3000 UHPLC system (Thermo Fisher) with a 50 min gradient from 99:1 to 60:40 buffer A:B ratio under basic pH conditions, and then consolidated into 18 super‐fractions. Each super‐fraction was then further separated by reverse phase XSelect CSH C18 2.5 μm resin (Waters) on an in‐line 150 × 0.075 mm column using an UltiMate 3000 RSLCnano system (Thermo Fisher). Peptides were eluted using a 60 min gradient from 98:2 to 60:40 buffer A:B ratio. Buffer A—0.1% formic acid, 0.5% acetonitrile, buffer B—0.1% formic acid, 99.9% acetonitrile, both buffers were adjusted to pH 10 with ammonium hydroxide for offline separation.

Eluted peptides were ionized by electrospray (2.2 kV) followed by mass spectrometric analysis on an Orbitrap Eclipse Tribrid mass spectrometer (Thermo Fisher) using multi‐notch MS3 parameters with real‐time search enabled. MS data were acquired using the FTMS analyzer in top‐speed profile mode at a resolution of 120 000 over a range of 375 to 1500 m/z. Following CID activation with a normalized collision energy of 35.0, MS/MS data were acquired using the ion trap analyzer in centroid mode and normal mass range. Using synchronous precursor selection, up to 10 MS/MS precursors were selected for HCD activation with a normalized collision energy of 65.0, followed by acquisition of MS3 reporter ion data using the FTMS analyzer in profile mode at a resolution of 50 000 over a range of 100–500 m/z. Proteins were identified and MS3 reporter ions quantified using maxquant (Max Planck Institute, Munich, Germany) against the UniprotKB database with a parent ion tolerance of 3 ppm, a fragment ion tolerance of 0.5 Da, and a reporter ion tolerance of 0.003 Da. Scaffold Q + S (proteome Software) was used to verify MS/MS‐based peptide and protein identifications [48].

Protein TMT MS3 reporter ion intensity values were assessed for quality using in‐house the proteinorm app, a user‐friendly tool for a systematic evaluation of normalization methods, imputation of missing values, and comparisons of different differential abundance methods [47]. Popular normalization methods were evaluated including log2 normalization (Log2), median normalization (Median), mean normalization (Mean), variance stabilizing normalization (VSN) [49], quantile normalization (Quantile) [50], cyclic loess normalization (Cyclic Loess) [51], global robust linear regression normalization (RLR) [52], and global intensity normalization (Global Intensity) [52]. The normalized data were used to perform statistical analysis using Linear Models for Microarray Data (limma) with empirical Bayes (eBayes) smoothing to the standard errors [51]. Proteins with an FDR‐adjusted *P*‐value < 0.05 and a fold change > 2 were considered significant.

### 2.12 Data processing and statistics

PA and PSM data were log‐transformed and uploaded to the perseus proteomics data analysis computational platform version 1.6.5.0 [53] to calculate *P*‐values and FDRs between groups: TurboABI1 #1 (*n* = 9) vs. TurboControl #1 (*n* = 9), TurboABI1 #1 (*n* = 9) vs. WT (*n* = 9). perseus two‐sample tests settings used were: one‐tailed (TurboABI1 #1‐sided) Student's *t*‐test, permutation‐based FDR; any other settings were left as program defaults (Table S4). Ratios were calculated by dividing group averages of untransformed PA or PSM. Collated PDL/MS data are available as Table S5. An rshiny app was developed to assist interpretation of TurboABI1 data based on retrospective statistical thresholding and pathway, interaction, and annotation database cross‐referencing (Table S6; https://maxpetersen.shinyapps.io/turboabi_data_ui_v2/).

### 2.13 Bioinformatics

GO biological process queries used david v6.8 [54, 55], with default settings for OFFICIAL_GENE_SYMBOL enrichment and background *Mus musculus* (Table S7). Known ABI1 and TAK1/MAP3K7 interactors were curated from Mint, IntAct, Biogrid, and StringDB databases (Table S8). Unless otherwise stated, StringDB interaction maps are presented with default interaction score (IS ≥ 0.4), where thicker edges indicate higher IS, and disconnected nodes are hidden.

### 2.14 Coimmunoprecipitation

WT NIH/3T3 grown to 70% confluency were washed twice with prewarmed PBS, then lysed on ice for 5 min with ice‐cold lysis buffer containing 25 mm Tris–HCl (pH 7.4), 150 mm NaCl, 5% glycerol, 1% NP‐40, and protease/phosphatase inhibitors. Cells were scraped, then collected lysates were passed 20 times through a 25.5 G needle, then five times through a 21.5 G needle. Lysates were cleared by centrifuging at 20 000× ***g*** for 10 min at 4 °C. 10% cleared lysate volume was collected for lysate analysis. Five microgram mouse IgG (Jackson ImmunoResearch, West Grove, PA, USA, 015‐000‐003) and 50 μL lysis‐buffer‐washed Protein G Dynabeads (Invitrogen, 10004D) were added to the remaining cleared lysate and incubated with rotation overnight at 4 °C. Supernatant was separated from beads using a magnet, then split evenly between two tubes for pulldowns. Five microgram mouse IgG1 isotype control (Cell Signaling Technology, Danvers, MA, USA, 5415S) was added to one tube, and 5 μg anti‐ABI1 (clone 1B9, MBL) to the other. Tubes were incubated with rotation overnight at 4 °C, then added to 50 μL washed Protein G beads and incubated with rotation overnight at 4 °C. Beads were then washed three times in wash buffer of the same composition as the lysis buffer above, except with 0.1% NP‐40. Sixty microliter sample reducing buffer was added to the beads, then samples were heated for 10 min at 98 °C. Supernatants were separated from beads using a magnet, then analyzed by western blot. Information about antibodies is available in Table S1.

### 2.15 TNFα stimulation

ABI1 deficient [Abi1(−/−) KO MEF cells: clone#3–6 (KO1), clone#3–11 (KO2) and Abi1(fl/fl) WT MEF cells, clone#3‐PP (KOC)]. Cells were a kind gift from Dr. Leszek Kotula [2]. These cells were derived from mouse embryos as described [2]. These cells were not authenticated by Short Tandem Repeat (STR) profiling. Cells were tested for mycoplasma upon receipt, when cell stocks are generated, and every 3 months on all actively growing cells using the MycoAlert mycoplasma Detection Kit (Lonza, LT07‐318) and DAPI staining. ABI1 overexpressing (OE) cells and ABI1 OE control (OEC) were TurboABI1 #1 and TurboControl #1 cell lines. 7.5e5 cells per condition were seeded in 10 cm plates and grown overnight in complete DMEM + P/S. Cells were washed once with prewarmed PBS, then 8 mL prewarmed complete DMEM or complete DMEM supplemented with 30 ng·mL^−1^ TNFα (Stemcell Technologies, Vancouver, BC, Canada, 78069.1) was added to each plate. Cells were incubated at 37 °C for 30 min or 12 h, then washed once with ice‐cold PBS. Two hundred and fifty microliter ice‐cold RIPA plus inhibitors was added to each plate and plates were incubated on ice for 5 min. Cells were scraped, then collected lysates were centrifuged at 20 000× ***g*** for 15 min at 4 °C. Cleared supernatants were prepared for SDS/PAGE. The experiment was repeated three times. Densitometry comparing ABI1 KO and ABI1 OE proteins was reported as a ratio to unstimulated control cells, normalized to GAPDH for RIPK1 and c‐CASP3, and further normalized to RIPK1 for RIPK1 phosphosites (*n* = 3 per protein).

For stimulation studies aimed at detecting phosphorylated TAK1, preparation conditions were like those described above, but 10 ng·mL^−1^ TNFα and 10 nm Calyculin A (Cell Signaling Technology, 9902S) were used for 15 min incubation with ABI1 KO and ABI1 KOC cells. Densitometry presented is from three replicate experiments, measured using imagej [43]. Densitometry from ABI1 KO MEF clones was averaged and compared to ABI1 KOC MEF clone per experiment, and statistical tests were performed on ABI1 KO experimental average vs. ABI1 KOC densitometry measurements using two‐tailed Student's *t*‐test. Information about antibodies is available in Table S1.

For stimulation assays in the presence of TNFR inhibitors, 100 ng·mL^−1^ TNFα was used. Takinib (MedChem Express, Monmouth Junction, NJ, USA, HY‐103490) was used at a final concentration of 100 nm, necrostatin (MedChem Express, HY‐15760) was used at a final concentration of 500 nm, and Z‐VAD (R&D Systems, Minneapolis, MN, USA, FMK001) was used at a final concentration of 10 μm. Annexin V orange dye (Sartorius, Goettingen, Germany, 4641) was used at a final dilution of 1:3200. The assay was performed on ABI1 KO and ABI1 KOC cells plated on 96‐well plates in quadruplicate. incucyte auto‐analyzer software was used to quantify confluency by phase and apoptosis by annexin V image‐integrated intensity. Annexin V was normalized to confluency per timepoint, and all timepoints were reported as a ratio of time = 0.

## 3 Results

### 3.1 Establishing and characterizing stable TurboID‐ABI1 and TurboID cell lines

**Fig. 1:**
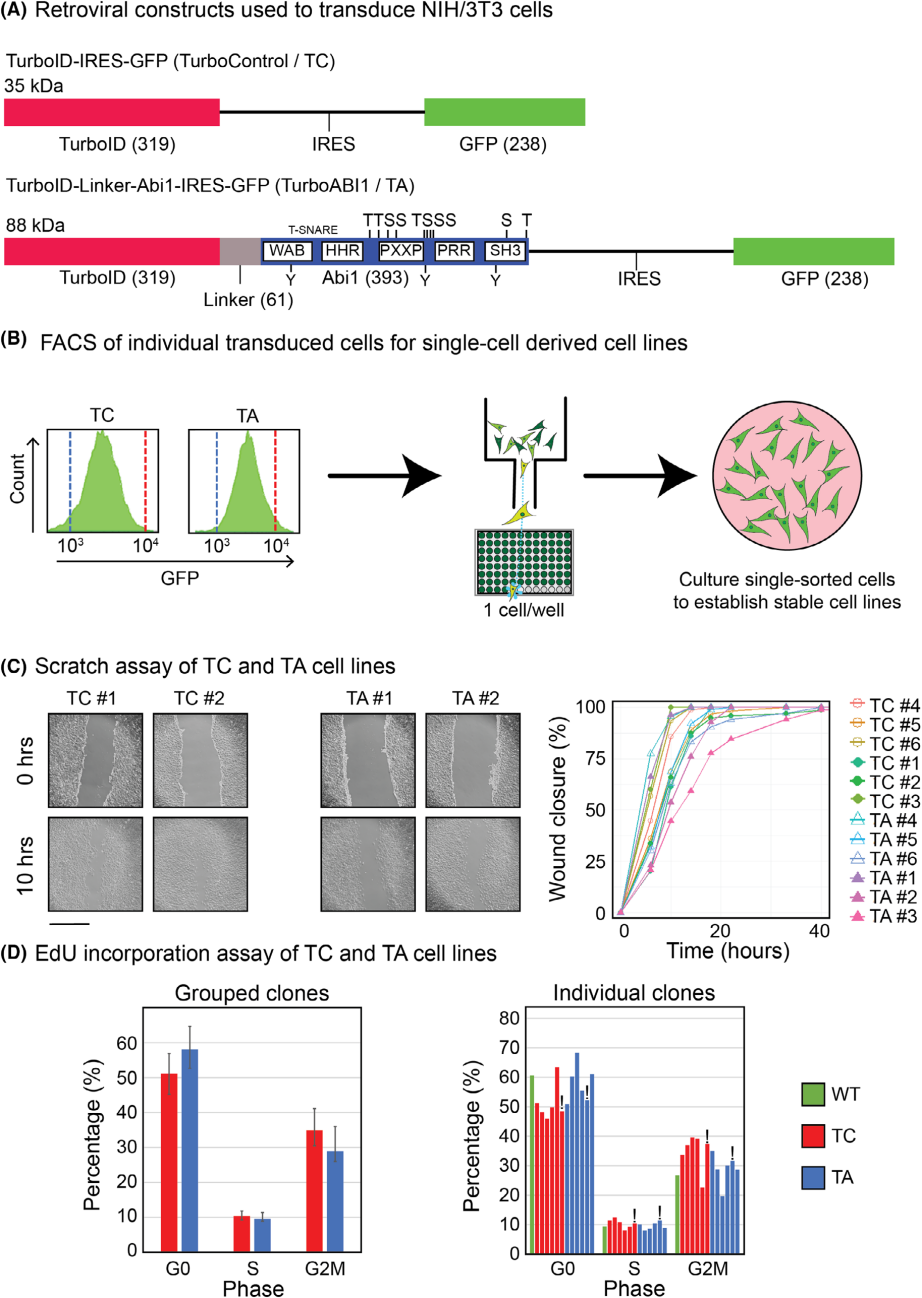
Tools and cell lines developed for Abelson interactor 1 (ABI1) proximity‐dependent labeling with biotin followed by mass spectrometry (PDL/MS). (A) Structure of murine stem cell virus (MSCV)‐TurboID‐internal ribosome entry site (IRES)‐green fluorescent protein (GFP; TurboControl/TC), and MSCV‐TurboID‐13 repeats of glycine–glycine–glycine–glycine‐serine (13xGGGGS) Linker‐ABI1‐IRES‐GFP (TurboABI1/TA) vectors used for retroviral transduction of NIH/3T3. (B) Left: Diagram representing single‐cell GFP fluorescence‐activated cell sorting (FACS) to establish single‐cell‐derived TurboABI1 and TurboControl NIH/3T3 cell lines. Right: Representative GFP flow cytometry histograms for FACS of TurboABI1 and TurboControl cell lines. The blue and red dashed lines are *x*‐axis markers to indicate the same GFP level among plots. (C) Left: Representative brightfield microscopy images of wound‐healing assay performed on TurboControl, and TurboABI1 cell lines, showing 0 and 10 h after scratching. The scale bar represents 890 μm. Right: Quantification of wound closure over time of TurboControl and TurboABI1 cell lines (*n* = 6). (D) Left: 5‐Ethynyl‐2′‐deoxyuridine (EdU) incorporation analysis of TurboControl and TurboABI1 clones (*n* = 6). Error bars represent standard deviation. Significance testing was performed using Student's *t*‐test. Right: EdU incorporation of individual clones of wildtype, TurboControl, and TurboABI1 cell lines. Bars marked with ‘!’ represent TurboControl #1 and TurboABI1 #1 clones used for PDL/MS.

To generate TurboID‐ABI1 (TurboABI1) and TurboID (TurboControl) retroviral constructs, we used the MSCV‐IRES‐GFP backbone for bicistronic GFP expression in mammalian cells (Tables S2 and S3). To mitigate encumbrance of the ABI1 C‐terminal SH3 domain, we designed the TurboABI1 retroviral construct so that TurboID was N‐terminal to murine ABI1 in the expressed fusion protein. To further avoid steric hindrance and to extend the labeling range, we included a linker comprising 13 repeats of glycine–glycine–glycine–glycine‐serine (13xGGGGS linker) [56] between TurboID and ABI1 (Fig. 1A). We transduced NIH/3T3 cells with MSCV‐TurboID‐Linker‐Abi1‐IRES‐GFP or MSCV‐TurboID‐IRES‐GFP retroviruses, followed by fluorescence‐activated cell sorting (FACS) by GFP (Fig. 1B and Fig. S1). Twelve cell lines were established from single‐sorted cells, six from TurboABI1 and six from TurboControl‐expressing groups.

To determine the phenotypic differences between TurboABI1 and TurboControl‐expressing cell lines, we assessed their wound‐healing capability by scratch assay over 40 h. We did not observe significant differences in wound closure between the TurboABI1 and TurboControl cell lines (Fig. 1C and Fig. S2). We also assessed cell cycle status by EdU incorporation assay and did not observe significant differences in cell cycle status between the TurboABI1 and TurboControl cell lines (Fig. 1D). Based on these results, we concluded that the single cell‐derived cell lines stably expressing biotin ligase or biotin ligase tagged ABI1 had similar growth phenotypes.

**Fig. 2:**
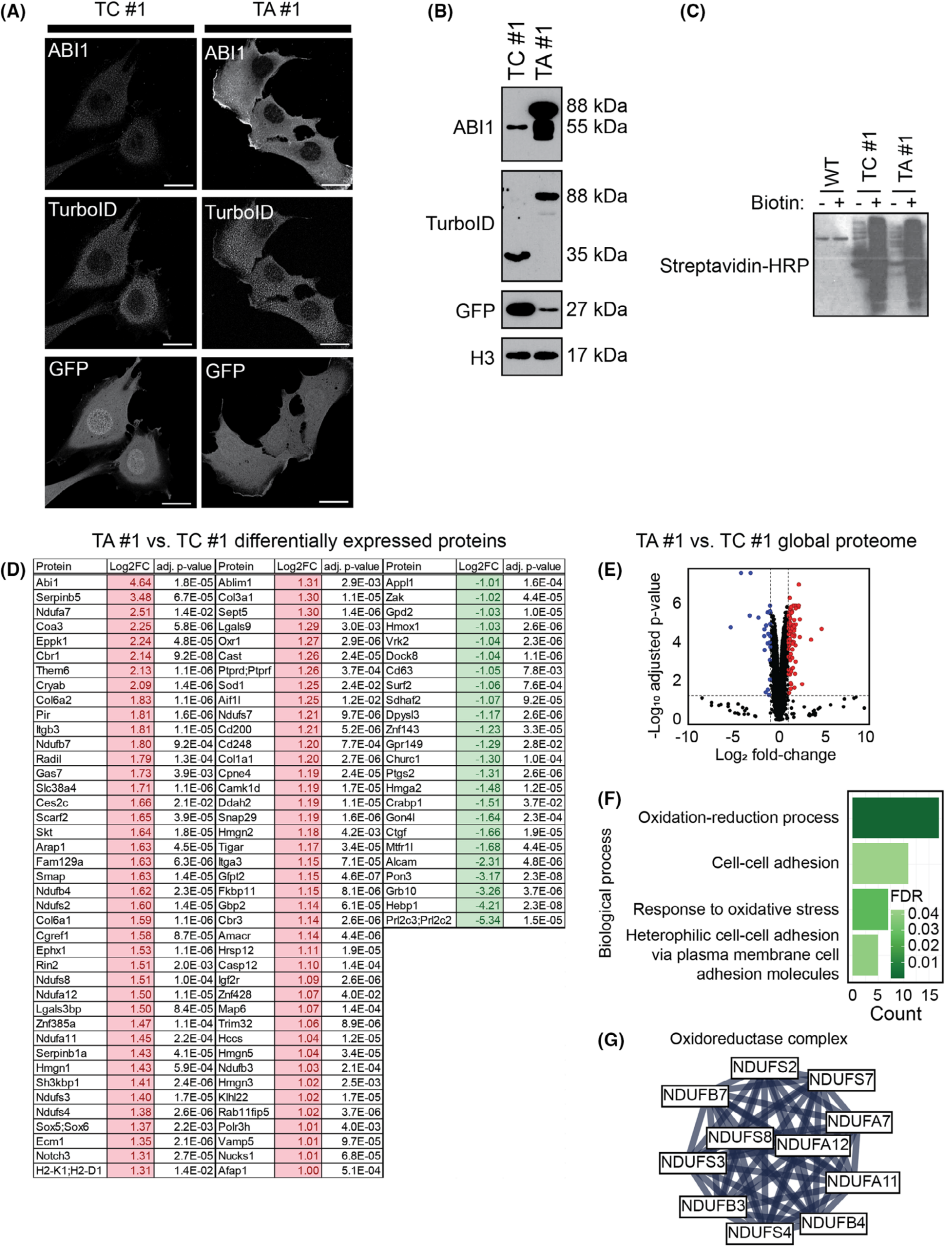
Characterization of NIH/3T3 cell lines used for proximity‐dependent labeling with biotin followed by mass spectrometry (PDL/MS) expressing TurboID (TurboControl #1) or TurboID linked to Abelson interactor 1 (ABI1; TurboABI1 #1). (A) Confocal immunofluorescence microscopy of TurboControl #1 and TurboABI1 #1 cells showing green fluorescent protein (GFP), TurboID, and ABI1 (*n* = 1). The scale bars represent 10 μm. (B) Representative expression levels of ABI1, TurboID, and GFP in TurboControl #1 and TurboABI1 #1 cell lines measured by immunoblotting (*n* = 3). (C) Western blot probed with streptavidin‐horseradish peroxidase (HRP) to detect biotinylated proteins in WT, TurboControl #1, and TurboABI1 #1 cell lines that were either uninduced or induced with 500 μm biotin for 10 min (*n* = 1). (D) List of proteins measured by global tandem mass tag (TMT) proteomics that are 2‐fold changed (adjusted *P*‐value ≤ 0.05) between uninduced TurboABI1 #1 and TurboControl #1 cell lysates (*n* = 3). (E) Volcano plot showing differentially expressed proteins in TurboControl #1 and TurboABI1 #1 cell lines. The vertical dashed lines, from left to right, correspond to Log_2_Fold‐Change (FC) of −1 and 1, respectively. The horizontal dashed line corresponds to adjusted *P*‐value 0.05. The points in color correspond to proteins listed in (A). Blue points are significantly downregulated in TurboABI1 #1 compared to TurboControl #1, and red points are significantly upregulated. (F) Gene Ontology (GO) biological process analysis of differentially expressed proteins measured by global proteomics, listed in (A), of uninduced TurboControl #1 and TurboABI1 #1 cell lines, showing processes identified with false discovery rate (FDR) ≤ 0.05. (G) Oxidoreductase interaction cluster derived from physical StringDB analysis, with interaction score set to 0.9 (highest confidence), of differentially expressed proteins measured from global TMT proteomics of uninduced TurboControl #1 and TurboABI1 #1, presented in (A).

The TurboABI1 #1 and TurboControl #1 clonal cell lines were selected for PDL/MS experiments. TurboID localization and expression in the two cell lines were similar as measured by immunofluorescence and immunoblotting (Fig. 2A,B). We observed increased biotinylation in TurboControl #1 and TurboABI1 #1 upon incubation with biotin, confirming the catalytic activity of TurboID. Wild type (WT) NIH/3T3 cells, as expected, did not show increased biotinylation upon biotin induction (Fig. 2C).

To determine the global effect of ABI1 overexpression in the TurboABI1 clone, we measured protein expression differences between TurboABI #1, TurboControl #1, and WT NIH/3T3 cell lines using Tandem Mass Tag (TMT) proteomics. We identified 106 proteins that showed a statistically significant (adjusted *P*‐value ≤ 0.05) expression level difference of 2‐fold or greater between the TurboABI1 #1 and TurboControl #1 cell lines. Of these 106 proteins, 82 were upregulated, and 24 were downregulated in TurboABI1 #1 cells compared to TurboControl #1 cells (Fig. 2D,E). Gene ontology (GO) biological process analysis of these 106 proteins identified significantly enriched processes of oxidation–reduction, cell–cell adhesion, and response to oxidative stress in TurboABI1 #1 when compared to TurboControl #1 cells (Fig. 2F). StringDB analysis of these proteins identified an interaction cluster associated with the oxidoreductase complex (Fig. 2D). The average log_2_FC values of ABI1 upregulation in the TurboABI1 #1 cell line were 4.8 and 4.6 in comparison to the TurboControl #1 and WT cell lines, respectively (Table S9). Based on these observations, we concluded that levels of ABI1 overexpression in the TurboABI1 clone, while modestly affecting the phenotype of the cells, would enable detection of proximal interactions that might otherwise remain below detection limits.

### 3.2 Proximity labeling experiments and quantitative analysis of ABI1 PDL/MS data

**Fig. 3:**
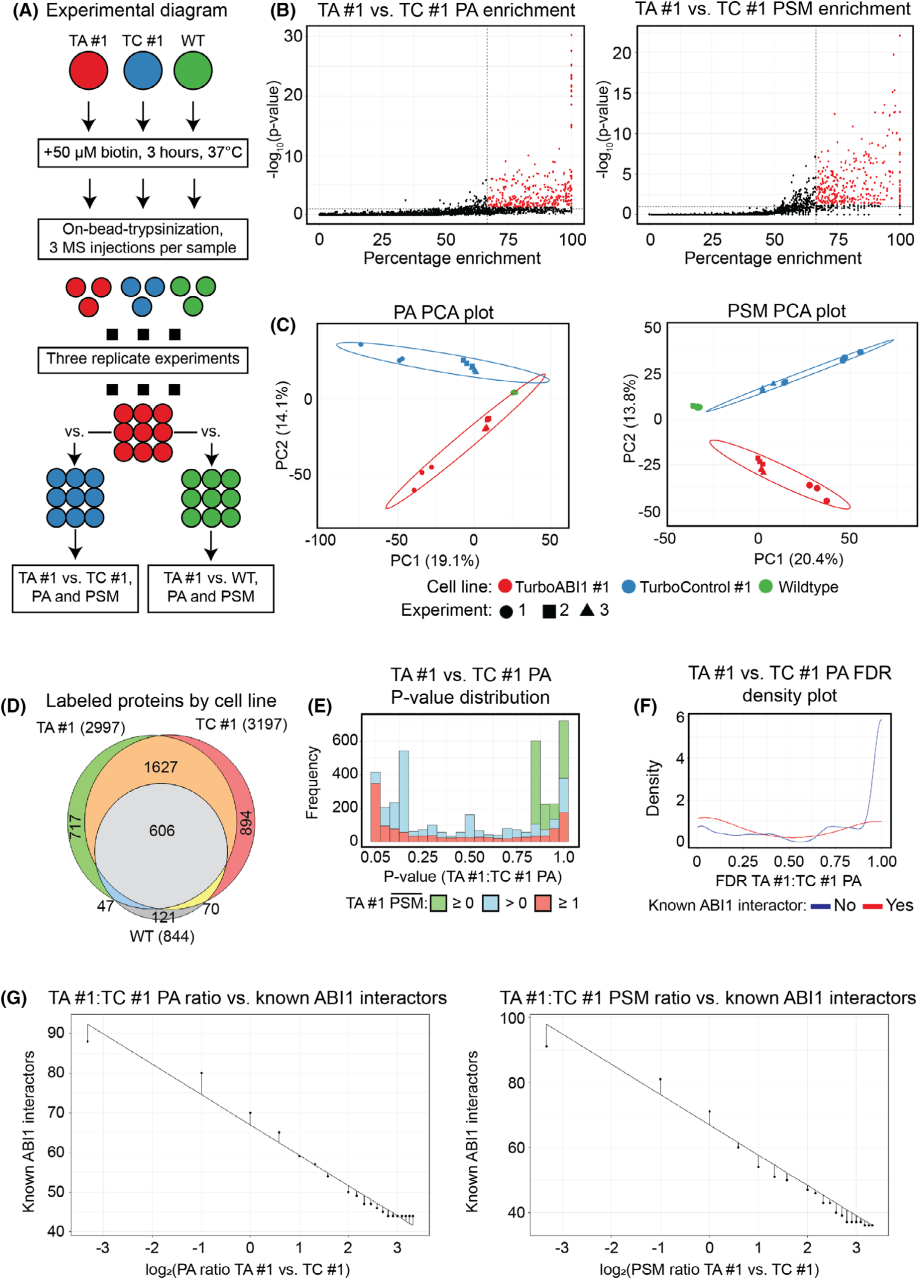
Quantitative analyses of proximity‐dependent labeling with biotin followed by mass spectrometry (PDL/MS) data from wild type (WT), TurboID‐expressing (TurboControl #1), and TurboID linked to Abelson interactor 1‐expressing (TurboABI1 #1) NIH/3T3 cell lines. (A) Experimental workflow describing cell lines used, experimental conditions, and biological and technical replicates resulting in 27 mass spectrometry (MS) runs. (B) One‐tailed TurboABI1 #1 vs. TurboControl #1 enrichment plots generated for peak area (PA; left) and peptide spectral match (PSM; right) measurements. Percentage enrichment, on the *x*‐axis, is calculated as (100*((TurboABI1 #1 peak area (PA) or peptide spectral match (PSM))/(TurboABI1 #1 + TurboControl #1 PA or PSM))), as an alternative to pseudocounts to include proteins not labeled by TurboControl High #1. The dashed lines are drawn at ratio and false discovery rate (FDR) thresholds defining ABI1‐proximally interacting hits (TurboABI1 #1 vs. TurboControl #1 ratio ≥ 1.5, FDR ≤ 0.05). Points in red represent proteins considered hits (*n* = 9). (C) Principal component analyses showing clustering by cell line and experiment of PA (left) and PSM (right) data. Points in red represent TurboABI1 #1, points in blue represent TurboControl #1, and points in green represent WT. Circles represent the first PDL/MS experiment, squares the second, and triangles the third. (D) Number of streptavidin‐enriched proteins identified per cell line. Overlaps indicate identification in multiple cell lines. (E) *P*‐value distribution of TurboABI1 #1 vs. TurboControl #1 PA ratio, subset by minimum average number of PSM per MS injection in TurboABI1 group (green: PSM ≥ 0, blue: > 0, red: ≥ 1). (F) Density plot of TurboABI1 #1 vs. TurboControl #1 PA FDR, subset by published evidence of ABI1 interaction curated from MINT, IntAct, StringDB, and Biogrid interaction evidence databases. (G) TurboABI1 #1 vs. TurboControl #1 PA (left) or PSM (right) log‐ratio vs. number of known ABI1 interactors. Vertical segments are drawn between data points and the best‐fit line was calculated by linear regression.

To establish the ABI1 interactome, we performed three independent PDL/MS experiments using TurboABI1 #1, TurboControl #1, and WT NIH/3T3 cell lines. We performed three technical replicate MS injections for each independent experiment, resulting in a total of 27 MS runs (Fig. 3A). To assure stringent data filtering, we used both protein peak area (PA; also known as protein ion intensity or abundance) and peptide spectral match (PSM) data for quantitative comparisons between TurboABI1 and controls. PA and PSM data both showed log‐normal distribution (Fig. S3); therefore, significance testing was performed on log‐transformed data. TurboABI1 measurements were compared to TurboControl and WT controls based on one‐tailed, TurboABI1‐sided significance tests to determine probable ABI1 interactors in the dataset (Fig. 3B). Principal component analyses indicated that PA and PSM data showed clustering by cell line and experiment (Fig. 3C). In three independent experiments, a total of 4082 unique proteins were identified, with 2997 proteins identified as enriched in the TurboABI1 #1 group (Fig. 3D). Taking into consideration only proteins identified with an average of 1 or more PSM per injection resulted in a higher proportion of targets with PA *P*‐value ≤ 0.05 for the TurboABI1 #1 vs. TurboControl #1 comparison (Fig. 3E). Interestingly, more known ABI1 interactors were identified within the group of targets with PA FDR ≤ 0.05 for TurboABI1 #1 vs. TurboControl #1 (Fig. 3F). Together, these data indicated an expected directionality of TurboABI1 #1 labeling while informing optimal FDR and average TurboABI #1 PSM thresholds. To determine optimal TurboABI1 #1 vs. TurboControl #1 PA and PSM ratios defining the most probable ABI1 proximal interactors, we plotted PA and PSM TurboABI1 #1:TurboControl #1 ratios vs. the number of known ABI1 interactors identified. For PA and PSM, a TurboABI1 #1 vs. TurboControl #1 ratio of 1.5 was close to the best‐fit line (Fig. 3G). This agreed with the TurboID/BioID field standard reporting cutoff of a 1.5‐fold enrichment. Labeling intensity in WT cell lines was negligible compared to TurboABI1 #1 and TurboControl #1 (Fig. S3). Considering these statistical interpretations, we defined the most probable ABI1 proximally interacting hits as follows: TurboABI1 #1 vs. controls PA ratio ≥ 1.5 (FDR ≤ 0.05), PSM ratio ≥ 1.5 (FDR ≤ 0.05), and minimum average 1 PSM per TurboABI1 #1 MS injection. A webapp that functions as a user interface to interpret this dataset under different statistical parameters was created and is available at https://maxpetersen.shinyapps.io/turboabi_data_ui_v2/ (GitHub: https://github.com/maxjohan/turboabi_data_ui, Table S6). We conclude from our analyses that utilization of both PA and PSM for quantitative MS data interpretation, in combination with analysis of MS data based on enrichment of known interactors, represents a stringent approach to establish PDL/MS‐based interactomes with high statistical confidence.

### 3.3 Known and new ABI1 interactors identified by PDL/MS

**Fig. 4:**
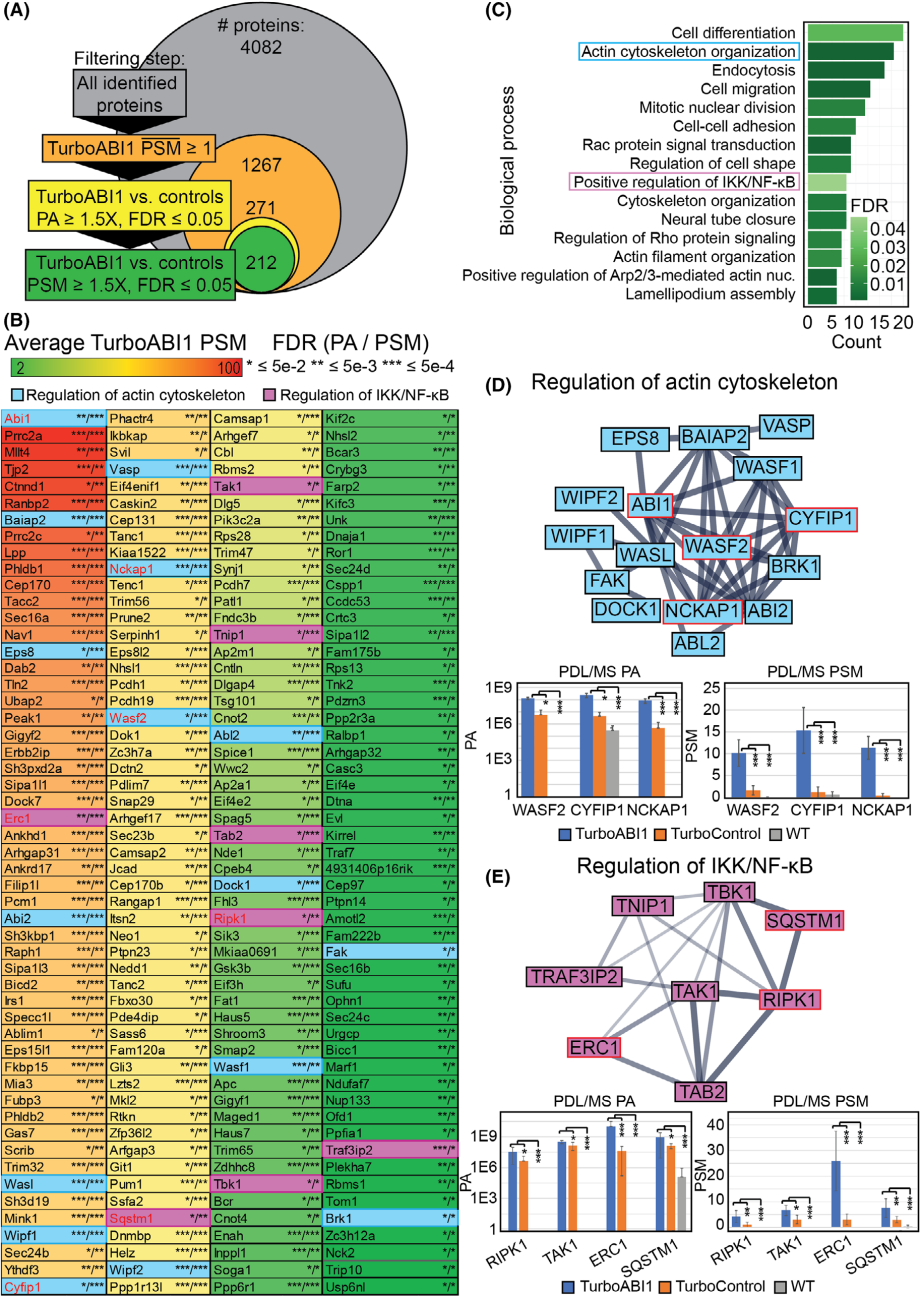
Analyses of significant Abelson interactor 1 (ABI1) proximally interacting proteins identified by proximity‐dependent labeling with biotin followed by mass spectrometry (PDL/MS) of wild type NIH/3T3 cells or NIH/3T3 cells expressing TurboID (TurboControl #1) or TurboID linked to ABI1 (TurboABI1 #1) identifies the role of ABI1 in tumor necrosis factor receptor (TNFR)‐transforming growth factor β‐activated kinase 1 (TAK1)‐nuclear factor kappa‐light‐chain‐enhancer of activated B cells (NF‐κB) signaling. (A) Data filtering strategy defining hits, or significant ABI1 proximally interacting proteins, and the number of proteins at each filtering step. (B) List of identified hits, sorted by average peptide spectral matches (PSMs) in TurboABI1 #1 group. Cells shaded in blue are associated with cytoskeleton organization, and cells shaded in purple are associated with inhibitor of nuclear factor kappa‐B kinase (IKK)/NF‐κB regulation indicated by Gene Ontology (GO) biological process analysis of hits. Proteins in red text were selected for further ABI1 interaction validation by coimmunoprecipitation. * = false discovery rate (FDR) ≤ 0.05, ** = FDR ≤ 0.005, *** = FDR ≤ 0.0005 for TurboABI1 #1 vs. TurboControl #1 peak area (PA) or PSM. (C) GO biological process analysis of hits, sorted by number of identified proteins assigned to each process and colored by GO biological process enrichment FDR. Boxed biological processes were selected for further validation by physical and functional experiments. (D) Top: StringDB interaction analysis of hits involved in cytoskeletal regulation, indicated by GO biological process analysis of hits. Thicker edges indicate a higher interaction score (0.4–0.9). Proteins outlined in red were selected for further validation by ABI1 coimmunoprecipitation. Bottom: PA and PSM measurements for indicated proteins from TurboABI1 #1, TurboControl #1, and wild type NIH/3T3 PDL/MS experiments (*n* = 9). Error bars represent standard deviation. * = FDR ≤ 0.05, ** = FDR ≤ 0.005, *** = FDR ≤ 0.0005. (E) Top: StringDB interaction analysis of hits associated with IKK/NF‐κB regulation, indicated by GO biological process analysis of hits. Thicker edges indicate a higher interaction score (0.4–0.9). Proteins outlined in red were selected for further validation by ABI1 coimmunoprecipitation. Bottom: PA and PSM measurements for indicated proteins from TurboABI1 #1, TurboControl #1, and wild type NIH/3T3 PDL/MS experiments (*n* = 9). Error bars represent standard deviation. * = FDR ≤ 0.05, ** = FDR ≤ 0.005, *** = FDR ≤ 0.0005.

Of the 4082 proteins identified by PDL/MS, 212 proteins met the aforementioned filtering criteria. As expected, ABI1 itself was the most labeled protein by average TurboABI1 PSM (Fig. 4B). Of the 212 proteins, 32 were known ABI1 interactors based on curated data in the StringDB, MINT, IntAct, and BioGRID interaction evidence databases (Table S8). Gene Ontology (GO) Biological Process (BP) enrichment analysis of the 212 ABI1 proximal interactors yielded both expected and unexpected significant (FDR ≤ 0.05) biological processes (BP). As anticipated, processes linked to actin cytoskeleton organization, including actin cytoskeleton organization, endocytosis, cell migration, cell–cell adhesion, actin filament organization or lamellipodium assembly were the most abundant within the top 15 hits. Surprisingly, positive regulation of IKK/NF‐κB signaling was detected as one of the top 15 hits (Fig. 4C). StringDB interaction mapping of ABI1 proximally interacting proteins showed, as expected, a cluster affecting cytoskeletal organization (Fig. 4D), as well as an unexpected cluster associated with IKK/NF‐κB signaling (Fig. 4E). The latter included TAK1 and TAK1‐interacting proteins. These analyses, considering the enrichment of known roles of ABI1, provided reasonable support that the unexpected GO BP enrichments were genuine. Because of our previous findings describing a role of ABI1 in regulating NF‐κB signaling in hematopoietic stem/progenitor cells from MPN patients,^24^ we sought to further characterize the role of ABI1 in TAK1‐mediated regulation of NF‐κB.

### 3.4 Identification of TAK1 and TAK1 associated proteins within the network of ABI1 proximal interactors

**Fig. 5:**
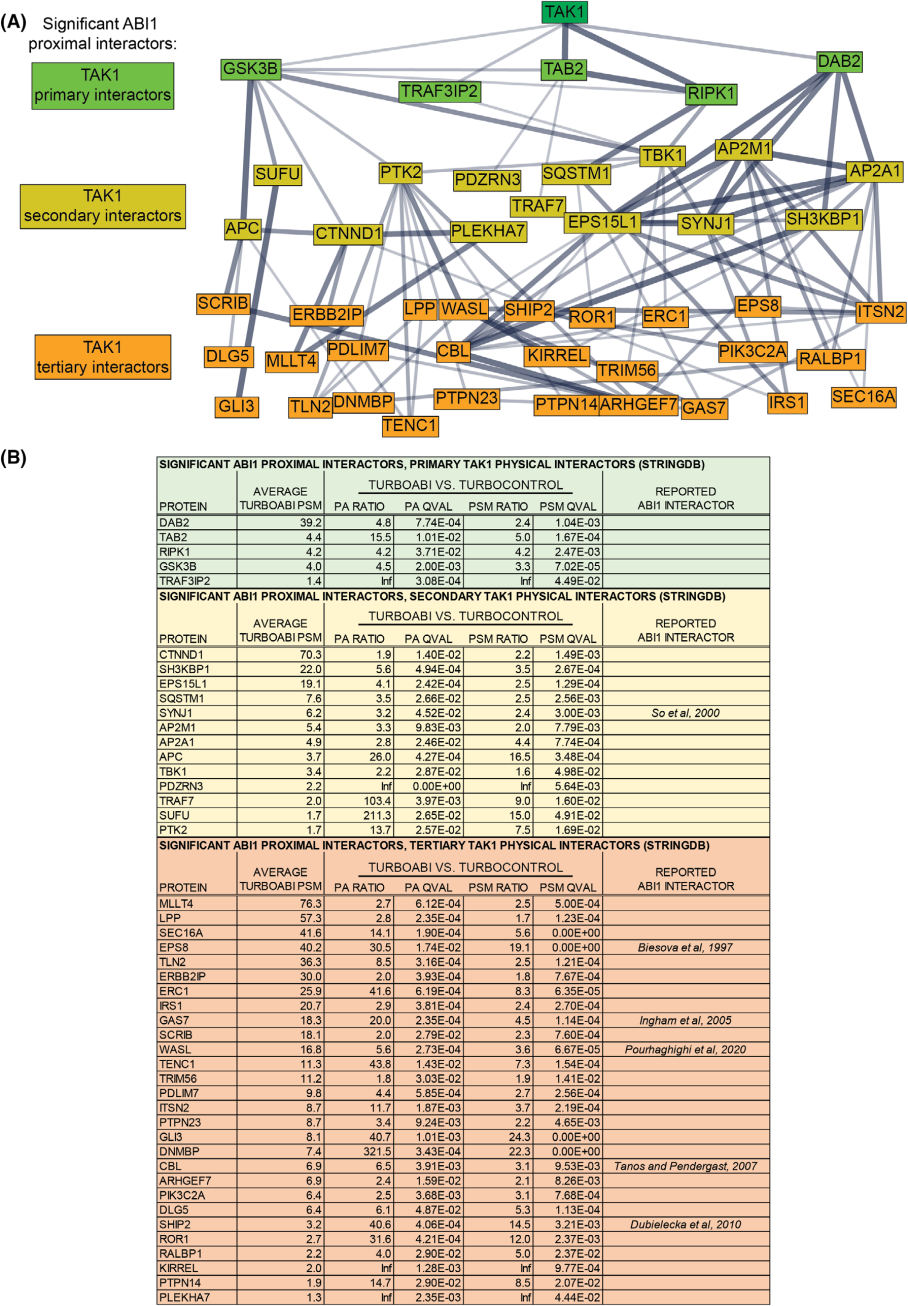
Bioinformatic analysis of significant proximal Abelson interactor 1 (ABI1) interactors shows relevance to transforming growth factor β‐activated kinase 1 (TAK1) interaction. (A) List of proximity‐dependent labeling with biotin followed by mass spectrometry (PDL/MS) data for statistically significant ABI1 proximal interactors that were also identified as primary, secondary, or tertiary TAK1 interactors by StringDB physical interaction mapping (interaction score [IS] ≥ 0.4). Hits are stratified by TAK1 interaction degree and sorted by average peptide spectral matches (PSM) identified in biotin‐stimulated cells expressing TurboID linked to ABI1 (TurboABI1 #1). (B) StringDB physical interaction map (IS ≥ 0.4) of statistically significant ABI1 proximal interactors that are also primary, secondary, or tertiary TAK1 interactors. TAK1 was manually input, and proteins were stratified by degree of TAK1 interaction. Proteins in green are primary TAK1 interactors, proteins in yellow are secondary TAK1 interactors, and proteins in orange are tertiary TAK1 interactors according to StringDB. Thicker edges indicate a higher interaction score.

To further explore ABI1 in the context of TAK1 regulation, we performed bioinformatic analysis to characterize the intersection between the measured ABI1 proximal interactome and the established TAK1 interactome curated from the StringDB, MINT, IntAct, and BioGrid interaction evidence databases. Of 227 curated TAK1 interactors (Table S8), ABI1 was found to proximally interact with six proteins: DAB2, TAK1, TAB2, RIPK1, GSK3B, and TRAF3IP2. Using StringDB, we identified 13 secondary TAK1 interactors and 28 tertiary TAK1 interactors that were also identified as significant ABI1 proximal proteins (Fig. 5A,B). This bioinformatic analysis suggested the role of ABI1 as a regulator of TAK1 activity and identified specific proteins that may be involved in ABI1‐associated regulation of TAK1.

### 3.5 ABI1 directly interacts with proteins involved in TAK1 signaling

**Fig. 6:**
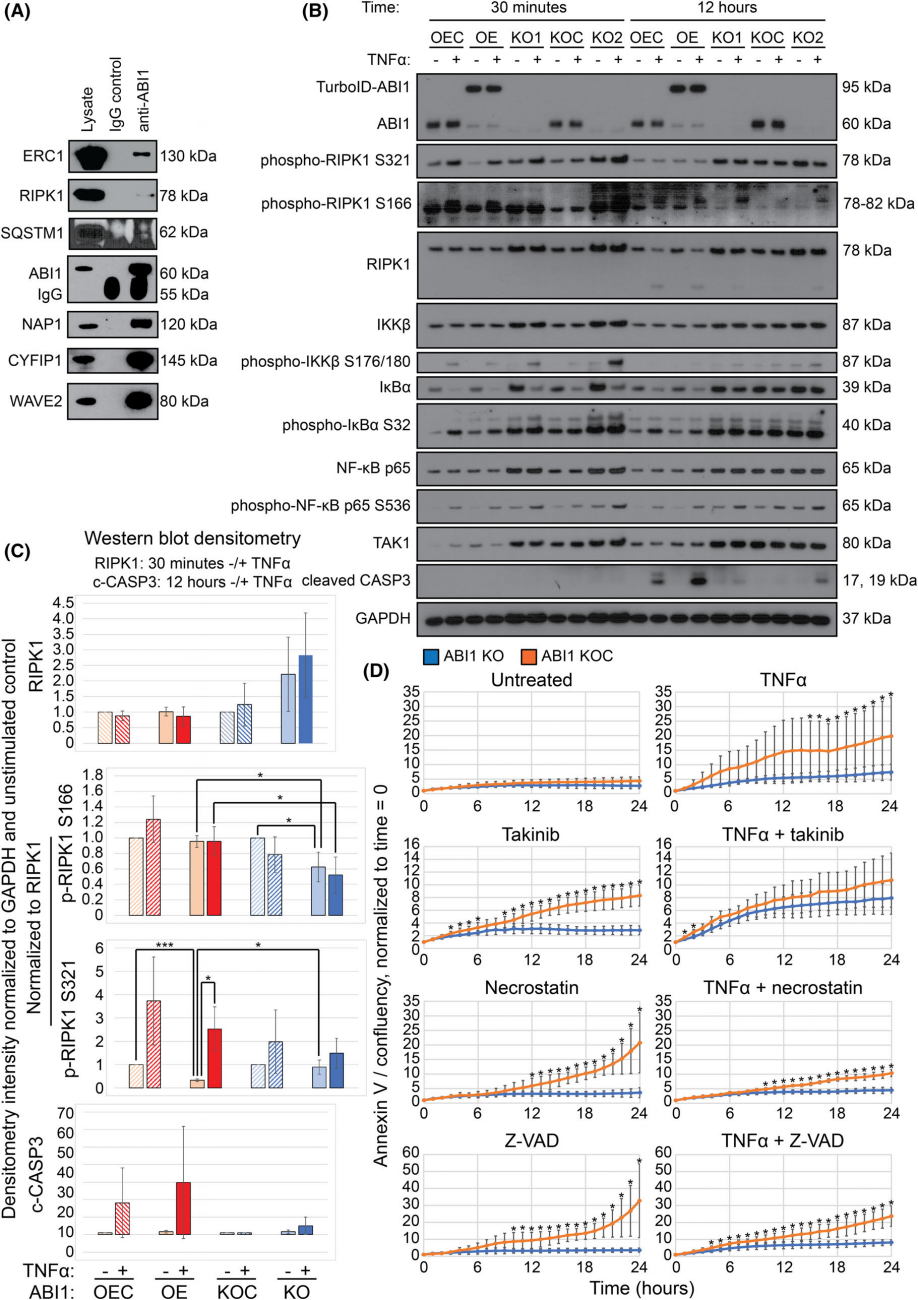
Abelson interactor 1 (ABI1) binds proteins involved in tumor necrosis factor receptor (TNFR) pathway signal transduction to affect receptor‐interacting serine/threonine‐protein kinase 1 (RIPK1) phosphorylation dynamics, caspase cleavage, and apoptosis. (A) Representative coimmunoprecipitations of NIH/3T3 lysates using mouse immunoglobulin G (IgG) or anti‐ABI1 to pull down selected significant ABI1 proximal interactors also involved in transforming growth factor β‐activated kinase 1 (TAK1)/nuclear factor kappa‐light‐chain‐enhancer of activated B cells (NF‐κB) pathway regulation and cytoskeleton organization (*n* = 3). (B) Western blots showing TAK1‐RIPK1‐NF‐κB pathway component and activation levels in tumor necrosis factor alpha (TNFα)‐stimulated ABI1 overexpressing (OE), ABI1 knockout (KO), and control (OEC and KOC, respectively) mouse embryonic fibroblast (MEF) cell lines (*n* = 3, see Fig. S5). Stimulations or non‐stimulations were performed for 30 min and 12 h. (C) Glyceraldehyde 3‐phosphate dehydrogenase (GAPDH) and unstimulated control‐normalized densitometry of Western blots for RIPK1, phospho‐RIPK1 S166, phospho‐RIPK1 S321, and cleaved caspase 3 (c‐CASP3) from lysates of ABI1 OE, OEC, KO, and KOC cells unstimulated and stimulated with TNFα for 30 min (RIPK1) or 12 h (c‐CASP3; *n* = 3). Phospho‐RIPK1 densitometries were further normalized to RIPK1 levels. Error bars represent standard deviation. *P*‐values were calculated using Student's *t*‐test. * = *P* ≤ 0.05, *** = *P* ≤ 0.0005. (D) TNFα stimulation of ABI1 KO and KOC cells in the presence of TNFR pathway inhibitors. Apoptosis over 24 h was measured using annexin V normalized to confluency, normalized to time = 0, using an Incucyte live‐cell imaging system and analysis software with imaging every hour. Error bars represent standard deviation (*n* = 4). *P*‐values were calculated using Student's *t*‐test. * = *P* ≤ 0.05.

Based on bioinformatic analysis of ABI1 proximal interactors that are also direct or indirect interactors of TAK1, we used coimmunoprecipitation to confirm the interaction between ABI1 and selected TAK1‐associated proteins in unstimulated WT NIH/3T3 cells. In selecting targets for the analysis, we prioritized TAK1‐associated proteins by strength of proximity labeling by TurboID‐ABI1 and by the fold‐enrichment relative to TurboID control cells (Fig. 4B). The results of the coimmunoprecipitation experiments supported a direct interaction between ABI1 and RIPK1, SQSTM1, and ERC1 (Fig. 6A). We confirmed the proximal labeling of ERC1 by TurboID‐ABI1 in a small‐scale biotinylation experiment followed by western blot of the whole cell and streptavidin‐enriched lysates (Fig. S4). We also analyzed for coimmunoprecipitation between ABI1 and TAK1, TNIP1, TAB2, and TBK1. The results supporting these interactions were not obtained. Based on ABI1 coimmunoprecipitations of TAK1‐interacting proteins, and because of the prominent roles TAK1 and RIPK1 play in regulating TNFR‐mediated balance of cell survival and death, we next focused on characterizing how ABI1 regulates TAK1 and RIPK1 in functional assays.

### 3.6 ABI1 dysregulation affects NF‐κB activation, RIPK1 phosphorylation status, and caspase cleavage

To measure the effect of ABI1 on TAK1‐RIPK1 signaling, we stimulated ABI1 overexpressing (OE) cells, ABI1 deficient (KO) cells, and the respective control MEF cell lines (OEC, KOC) with TNFα for 30 min and 12 h. We then assessed the TAK1‐RIPK1‐NF‐κB pathway activation status by immunoblotting in three independent experiments (Fig. 6B,C and Fig. S5). We observed general, independent of TNFα stimulation length, elevation of TAK1, RIPK1, and NF‐κB pathway components including IKKβ, IκBα, and NF‐κB p65 and their phosphorylated species in ABI1 KO cells compared to ABI1 OE and their control cells (Fig. 6B and Fig. S5). After 30 min of TNFα stimulation, we noted increased levels of RIPK1 in ABI1 KO cells compared to ABI1 KOC, OE, and OEC cells, regardless of stimulation status, although this measurement did not reach statistical significance (Fig. 6B,C). RIPK1‐normalized RIPK1 S166 phosphorylation status upon 30 min TNFα stimulation, indicative of RIPK1 autoactivation prior to apoptosis [57], was largely unchanged in ABI1 OE cells compared to ABI1 OEC, but decreased in ABI1 KO cells compared to ABI1 KOC and to ABI1 OE. Without stimulation, ABI1 KO showed 1.6‐fold significantly less RIPK1 S166 phosphorylation than ABI1 KOC, and 1.5‐fold significantly less than ABI1 OE cells. With stimulation, ABI1 KO showed 1.8‐fold significantly less RIPK1 S166 phosphorylation than ABI1 OE cells (Fig. 6B,C and Fig. S5). RIPK1 normalized RIPK1 S321 phosphorylation, indicative of inhibition of apoptosis [58, 59], showed a 3.0‐fold decrease in ABI1 OE compared to control after 30 min without stimulation and was decreased, but did not reached statistical significance after 30 min of stimulation. RIPK1 normalized RIPK1 S321 phosphorylation levels were not significantly affected in ABI1 KO compared to control after 30 min with or without TNFα stimulation (Fig. 6B,C and Fig. S5). Compared to unstimulated conditions, ABI1 OE showed 7.6‐fold significantly increased RIPK1‐normalized RIPK1 S321 phosphorylation (ABI1 OEC showed 3.7‐fold, *P* = 0.11), and ABI1 KO showed a 1.7‐fold increase, although this was not significant (ABI1 KOC showed a 2‐fold increase, *P* = 0.28; Fig. 6C). ABI1 KO showed 2.7‐fold significantly more RIPK1‐normalized RIPK S321 phosphorylation than ABI1 OE without stimulation, and 1.7‐fold less after 30‐min stimulation, although this did not meet statistical significance (*P* = 0.13; Fig. 6C). In congregation, these data indicated decreased levels of proapoptotic phospho‐RIPK1 S166 in ABI1 KO cells compared to ABI1 OE and controls, and elevated levels of RIPK1 and phospho‐RIPK S321 in ABI1 KO cells, linking loss of ABI1 to decreased susceptibility to TNFα‐induced apoptosis. To further assess the role of ABI1 in modulating apoptosis, we examined the status of caspase 3 cleavage upon TNFα exposure. ABI1 OE cells showed elevated caspase 3 cleavage compared to control after 30 min or 12 h stimulation with or without TNFα stimulation, whereas ABI1 KO showed only modestly elevated caspase 3 cleavage compared to control after 12 h with TNFα stimulation, although no statistical significance was observed among ABI1 OE, KO, and control cell lines (Fig. 6B,C and Fig. S5). These data suggest resistance to TNFα‐induced apoptosis in ABI1 KO cells, and support the interpretation that elevated activity of IKK/NF‐κB survival signaling and increased RIPK1‐dependent antiapoptotic signaling is present in ABI1 KO cells.

### 3.7 ABI1 deficiency protects cells from TNFα‐mediated apoptosis, dependent on TAK1 activity

To further characterize the effect of ABI1 deficiency on TNFα‐mediated apoptosis, we stimulated ABI1 KO and KOC cells with TNFα in the presence of TNFα pathway inhibitors and measured apoptosis using the apoptotic marker annexin V (Incucyte Annexin V Orange Dye) and an Incucyte live‐cell imaging system. We used the TAK1 autophosphorylation inhibitor takinib (at 100 nm [60]), the RIPK1 S166 autophosphorylation inhibitor necrostatin (at 500 nm [61]), and the pan‐caspase inhibitor Z‐VAD (at 10 μm [62]). Without stimulation, ABI1 KO and KOC cells showed similar levels of confluency‐normalized apoptosis over a 24 h period (Fig. 6D). After 15 h of TNFα stimulation, ABI1 KO cells showed a statistically significant 3‐ to 4‐fold reduction in apoptosis compared to KOC cells (Fig. 6D). In the presence of takinib alone, ABI1 KO cells showed significantly less cell death than KOC cells over 24 h (Fig. 6D). However, in the presence of TNFα and takinib, both KO and KOC cells showed similarly increased levels of apoptosis compared to unstimulated conditions (Fig. 6D), indicating that ABI1 acts downstream of TAK1 upon TNFα stimulation. In the presence of necrostatin, Z‐VAD, and necrostatin or Z‐VAD with TNFα, ABI1 KO cells showed significantly less apoptosis than KOC cells (Fig. 6D), indicating that loss of ABI1 affects communication between TAK1 and RIPK1 and RIPK1 and caspases. Together, these data suggest that ABI1 deficiency protects cells from TNFα‐mediated cell death, and this protection is decreased upon TAK1 inhibition.

## 4 Discussion

The use of proximity‐dependent labeling with biotin by TurboID combined with stringent analysis of MS data allowed us to identify a previously unrecognized role of ABI1 in regulating TAK1‐RIPK1 signaling. We attained high depth proximity labeling and detection by using large numbers of cells in replicate experiments with stable TurboID‐ABI1 and TurboID control single‐cell‐derived cell lines that expressed similar levels of TurboID ligase. Additionally, since MS‐generated PA and PSM measurements are uniquely affected by protein properties [56, 63, 64], we considered both metrics to identify ABI1 proximal interactors with high confidence. We developed the ‘TurboAbi data UI’ program to assist interpretation of proximity labeling data, based on both quantitative analysis and cross‐referencing to bioinformatic databases.

**Fig. 7:**
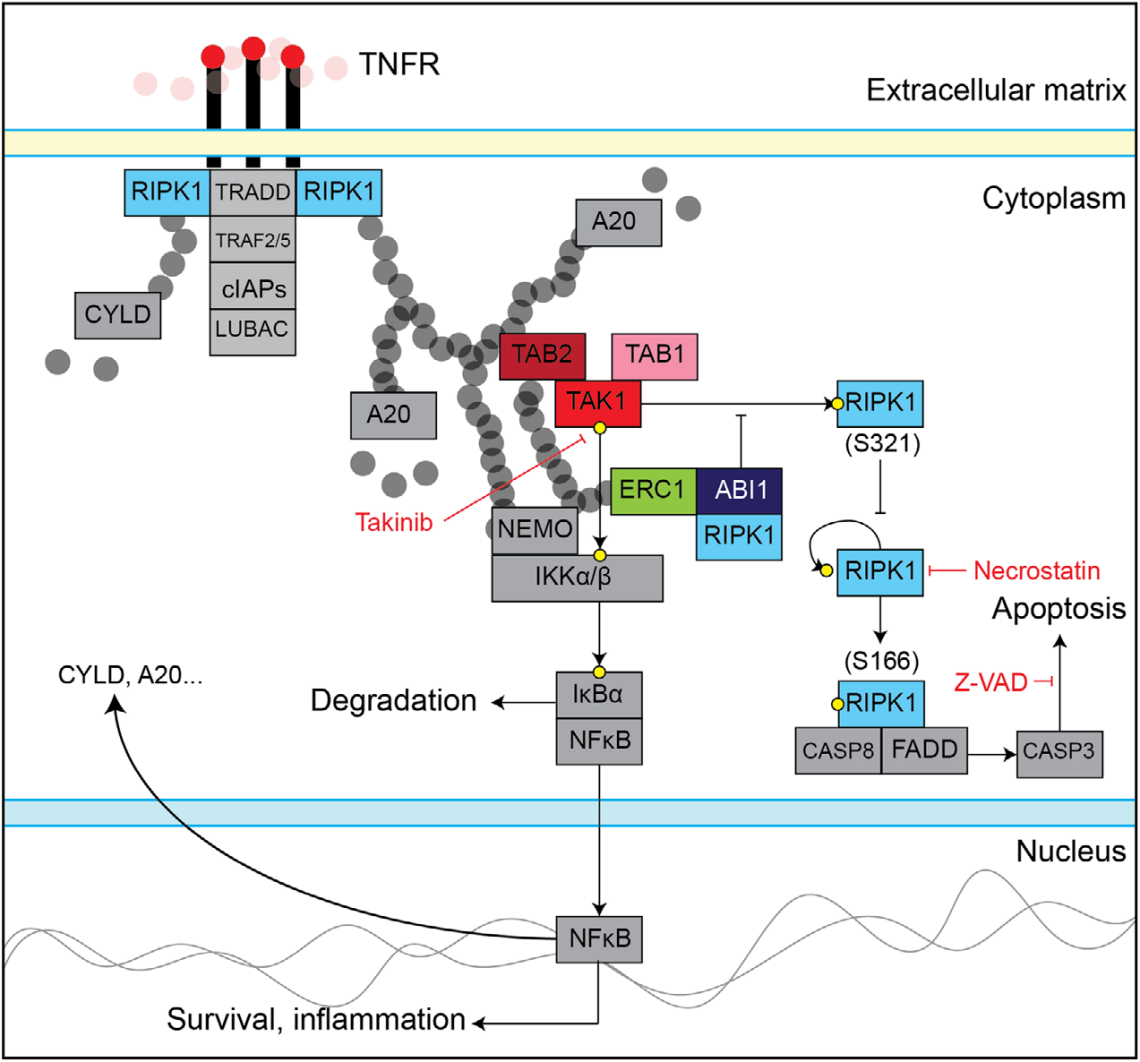
Diagram showing proposed Abelson interactor 1 (ABI1) role in tumor necrosis factor receptor (TNFR)‐transforming growth factor β‐activated kinase 1 (TAK1)‐receptor‐interacting serine/threonine‐protein kinase 1 (RIPK1) signaling. Colored boxes represent proteins labeled as significant ABI1 proximal interactors. Gray boxes represent proteins not significantly labeled as ABI1 proximal interactors. Red circles represent tumor necrosis factor alpha (TNFα). Gray circles represent ubiquitin. Yellow circles represent phosphorylation.

TAK1 is known to control cell viability and inflammation by activating downstream effectors such as NF‐κB, but also through NF‐κB‐independent pathways including a RIPK1 signaling axis activated in response to TNFα or IL‐1 [65]. RIPK1 is found both bound to the activated TNFR complex and uncoupled within the cytoplasm [30, 59, 66, 67, 68]. In our dataset, we did not identify TNFR complex I‐associated proteins such as TRADD, TRAFs, or cIAP1/2 as ABI1 proximal interactors. Instead, we identified TAK1, TAB2, ERC1, PPP6C, and TBK1, all of which are known to proximally interact with TAK1 bound to TNFR complex I‐based RIPK1‐linked polyubiquitin chains. Based on these observations, we hypothesize that the placement of the ABI1‐RIPK1 interaction is not at the TNFR under‐membrane assembly site but downstream of TAK1 (Fig. 7).

In addition to regulation of membrane‐proximal RIPK1 by ubiquitination and deubiquitination, phosphorylation of both membrane‐proximal and cytosolic RIPK1 influences the balance between TNFR‐mediated cell survival and death. RIPK1 is both autophosphorylated and phosphorylated by other kinases, RIPK1 itself being the only known RIPK1 kinase substrate [57]. While around 40 RIPK1 phosphosites have been recorded [69], RIPK1 S166 phosphorylation is accepted as the principal biomarker of RIPK1 cell death‐promoting activity [57]. RIPK1 S166 autophosphorylation opposes TAK1‐mediated survival signaling by initiating a caspase cleavage cascade leading to apoptosis. However, RIPK1 S166 phosphorylation alone is not sufficient to induce cell death [70], indicating the importance of other RIPK1 phosphosites and protein interactions. RIPK S321 phosphorylation is associated with decreased RIPK1 S166 phosphorylation and decreased cell death, preventing complex IIb formation but not affecting NF‐κB signaling [58, 59]. The mechanism of RIPK1 S321 phosphorylation is unclear, as RIPK1 S321 was shown by Geng *et al*. [58] to be phosphorylated by TAK1 in response to TNFα stimulation, and by Jaco *et al*. [59] to be dependent on a TNFR/LPS‐induced phosphorylation cascade involving TAK1, p38, and MK2. RIPK1 plays a dual role in TNFR signaling, acting as a structural element upholding complex I formation and prosurvival signaling through NF‐κB activation, independent of RIPK1 kinase activity, and as a promoter of cell death through autophosphorylation and caspase activation. A better understanding of RIPK1‐dependent regulation seemingly holds the key to understanding TNFR‐regulated balance of survival and death.

Our results indicated decreased RIPK1 S321 phosphorylation in unstimulated ABI1 overexpressing cells, which is maintained upon TNFα stimulation, suggestive of sequestration and blockade of free RIPK1 by ABI1 and an overall negative regulatory effect of ABI1 on RIPK1 S321 phosphorylation and its associated antiapoptotic signal. In ABI1 KO cells, we detected elevated levels of TAK1‐NF‐κB pathway components in steady state, including RIPK1, and consistently elevated NF‐κB pathway activation upon TNFα stimulation, in agreement with a prosurvival signal. Additionally, we observed decreased cell death in ABI1 KO compared to control cells upon TNFα stimulation, which was not observed in response to TAK1 inhibition. In our assay conditions we were not able to detect active TAK1, autophosphorylated on Thr184/187, in response to 30 min or 12 h TNFα stimulation. However, using phosphatase inhibitor calyculin A in combination with TNFα [71, 72], we observed increased TAK1 activation in ABI1 KO compared to control cells after a 15 min stimulation (Fig. S6), which may be linked to elevated baseline levels of TAK1 and RIPK1, consistent with more active S321 phosphorylation of RIPK1 by TAK1. This interpretation is consistent with high caspase cleavage observed in stimulated ABI1 OE cells, while the ABI1 absence appeared to provide an apoptosis protection effect by comparison. Furthermore, downstream inhibition of phospho‐RIPK1 S166 using necrostatin, or caspase cleavage using Z‐VAD, maintained the apoptosis protection observed in ABI1 KO compared to control cells. Together, this suggests that the apoptosis protection observed in ABI1 KO cells is granted by increased total phospho‐RIPK1 S321, through a TAK1‐dependent mechanism that primes cells for protection against TNFα‐induced death by depleting the RIPK1 pool available for S166 autophosphorylation and subsequent apoptosis, enabling prosurvival NF‐κB activation. Overall, these data suggest that ABI1 represses antiapoptotic signaling by sequestering RIPK1 to attenuate S321 phosphorylation by TAK1 (proposed model shown in Fig. 7), supporting a role for ABI1 in TAK1‐RIPK1‐NF‐κB‐mediated balance of death and survival.

ABI1 loss in hematopoietic stem/progenitor cells was previously shown to be associated with development of a myeloproliferative neoplasm. This association was linked to elevated NF‐κB signaling [24], prompting the question of how ABI1 mechanistically affects NF‐κB signaling to promote a malignant cell phenotype. In the present study, using proximity‐dependent labeling, we identified proximal interactions between ABI1 and components of the TNFR‐NF‐κB signaling pathway, including TAK1 and RIPK1. Based on our findings, we conclude that increased antiapoptotic RIPK1 phosphorylation, mediated by TAK1, offers a mechanistic link to sustained NF‐κB prosurvival signaling and resistance to RIPK1‐dependent cell death in ABI1 deficient cells.

## 5 Conclusion

Decreased ABI1 expression was found in hematopoietic stem/progenitor cells in patients with myeloproliferative neoplasm (MPN), and murine bone marrow‐targeted depletion of ABI1 was shown to be associated with an MPN‐like phenotype mechanistically linked to activation of SFKs, STAT3, and NF‐κB pathways [24]. We used proximity‐dependent labeling to detail mechanistic links between ABI1 and SFKs, STAT3, and NF‐κB and uncover details of involvement of ABI1 in cancer‐linked signaling pathways. We identified proximal interactions between ABI1 and components of the TNFR‐NF‐κB signaling pathway, and we uncovered that increased antiapoptotic RIPK1 phosphorylation, mediated by TAK1, constitutes a mechanistic link to sustained NF‐κB prosurvival signaling and resistance to RIPK1‐dependent cell death in ABI1‐deficient cells. Our proximity‐dependent labeling‐based strategy enabled mapping of the ABI1 proximal interactome, revealing a previously unknown role of this adaptor protein in TAK1/RIPK1‐based regulation of cell death and survival.

## Conflict of interest

The authors declare no conflict of interest.

## Author contributions

Conceptualization, investigation, and data analysis MP, PMD, AC, MPa, AR, NA, JM, PG, LK, OL, TZ, and PB. Writing—Original Draft, MP; Writing—Review & Editing, MP, PMD, PG, LK, MPa, OL, TZ, and PB; Software, MP; Visualization, MP; Project Administration, PMD. Data Curation, MP. All the authors read and approved the final version of the article.

### Peer review

The peer review history for this article is available at https://publons.com/publon/10.1002/1878‐0261.13374.

## Supporting information


**Table S1.** Vectors, primers, antibodies, and stains used in this study.

**Table S2.** Vector map of MSCV‐TurboID‐IRES‐GFP. Sequencing primers and contiguous sequences read by Sanger sequencing are included in Snapgene.

**Table S3.** Vector map of MSCV‐TurboID‐Linker‐Abi1‐IRES‐GFP. Sequencing primers and contiguous sequences read by Sanger sequencing are included in Snapgene.

**Table S4.** Perseus file used to calculate *P*‐values and FDRs for PDL/MS data.

**Table S5.** Collated TurboABI1 MEF PDL/MS data.

**Table S6.** TurboAbi data UI R code.

**Table S7.** DAVID analyses of significant proteins measured by TurboABI1 MEF PDL/MS.

**Table S8.** Known ABI1 and TAK1/MAP3K7 interactors, curated from StringDB, MINT, IntAct, and BioGRID interaction databases.

**Table S9.** Global TMT proteomics data of uninduced WT, TurboControl #1, and TurboABI1 #1 cell lysates (*n* = 3).

**Fig. S1.** Flow cytometry to detect GFP expression in WT, TurboControl, or TurboABI1 cell lines.

**Fig. S2.** Wound healing assay of TurboControl and TurboABI1 cell lines.

**Fig. S3.** Lognormality of PSM and PA data generated from labeling and MS of TurboABI1 #1, TurboControl #1, and NIH/3T3 WT cell lines.

**Fig. S4.** Small scale biotin labeling of induced TurboControl and TurboABI1 cell lines.

**Fig. S5.** Western blots of NF‐κB pathway components and activation in ABI1 OE, KO, and control cell lines stimulated with TNFα.

**Fig. S6.** Western blots of ABI1 KO and WT cell lines stimulated with TNFα and Calyculin A.

## Data Availability

Data supporting findings of this study, including processed mass spectrometry data and quantitative and bioinformatic analyses, are available in Tables [Link], [Link]. Known ABI1 and TAK1 interactors are available in Table S8 and were derived from the following resources available in the public domain: STRINGdb (https://string‐db.org), BioGRID (https://thebiogrid.org), The Molecular INTeraction Database (https://mint.bio.uniroma2.it), and IntAct (https://www.ebi.ac.uk/intact/home). An rshiny app was developed to assist interpretation of TurboABI1 data based on retrospective statistical thresholding and pathway, interaction, and annotation database cross‐referencing (Table S6; https://maxpetersen.shinyapps.io/turboabi_data_ui_v2/).
