# Supplementary figures and images for: Proximity proteomics reveals role of Abelson interactor 1 in the regulation of TAK1/RIPK1 signaling
Source: Mol Oncol. 2023 May 12;17(11):2356–79. doi: 10.1002/1878-0261.13374 (PMC10620119; doi:10.1002/1878-0261.13374)

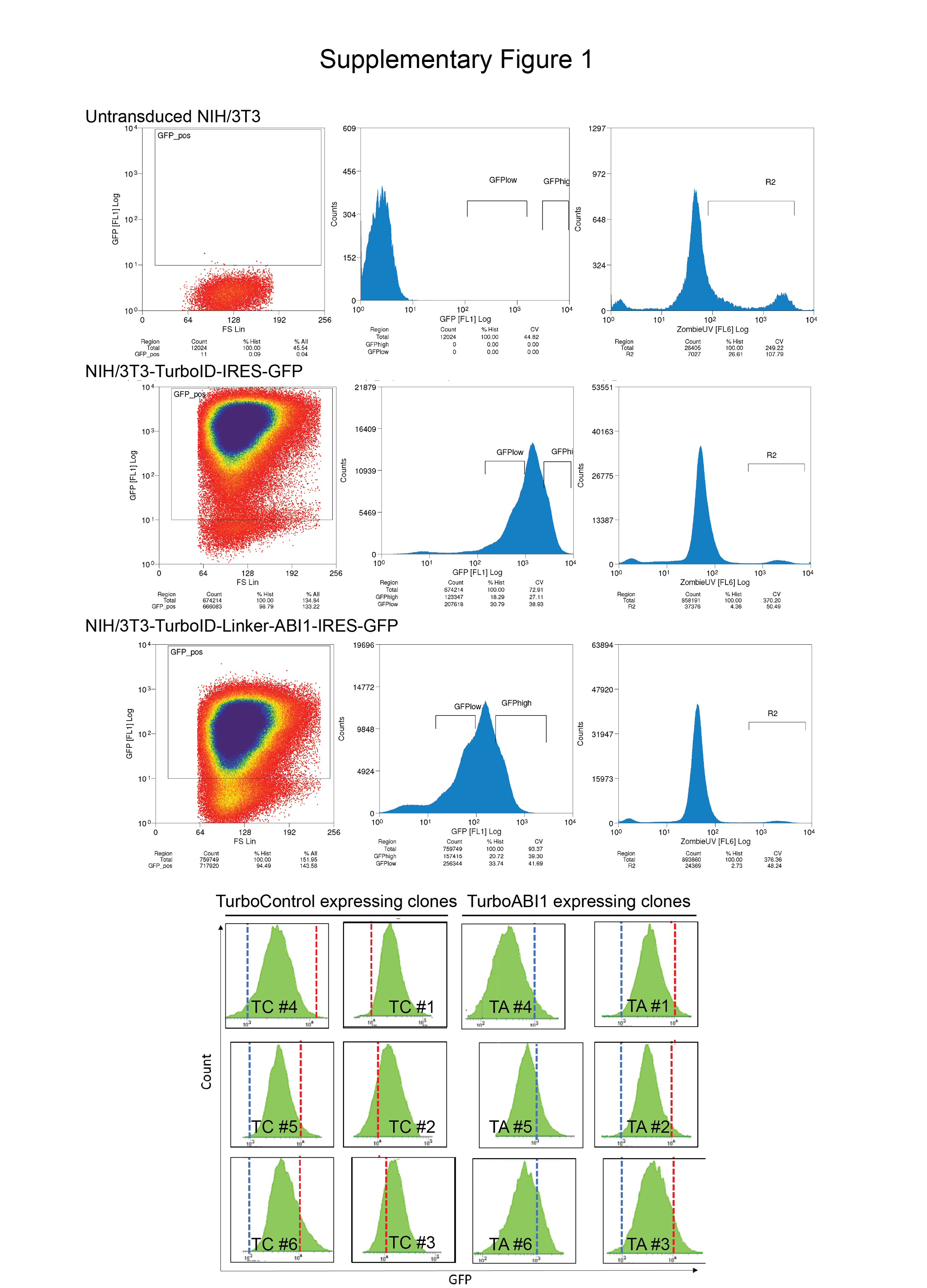

Supplement: Supplementary file 10 — Fig. S1. Flow cytometry to detect GFP expression in WT, TurboControl, or TurboABI1 cell lines. [file MOL2-17-2356-s001.tif]

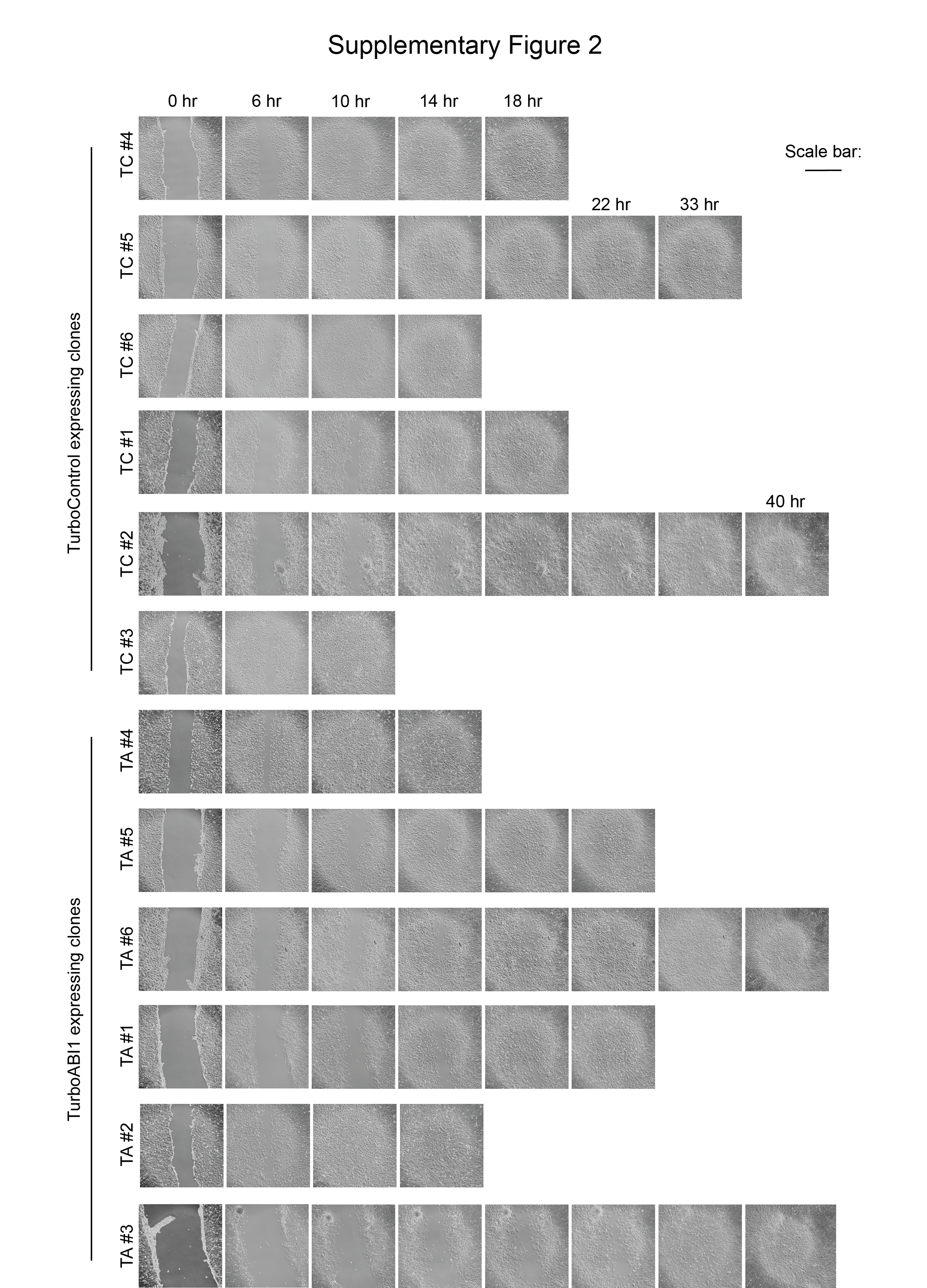

Supplement: Supplementary file 11 — Fig. S2. Wound healing assay of TurboControl and TurboABI1 cell lines. [file MOL2-17-2356-s007.tif]

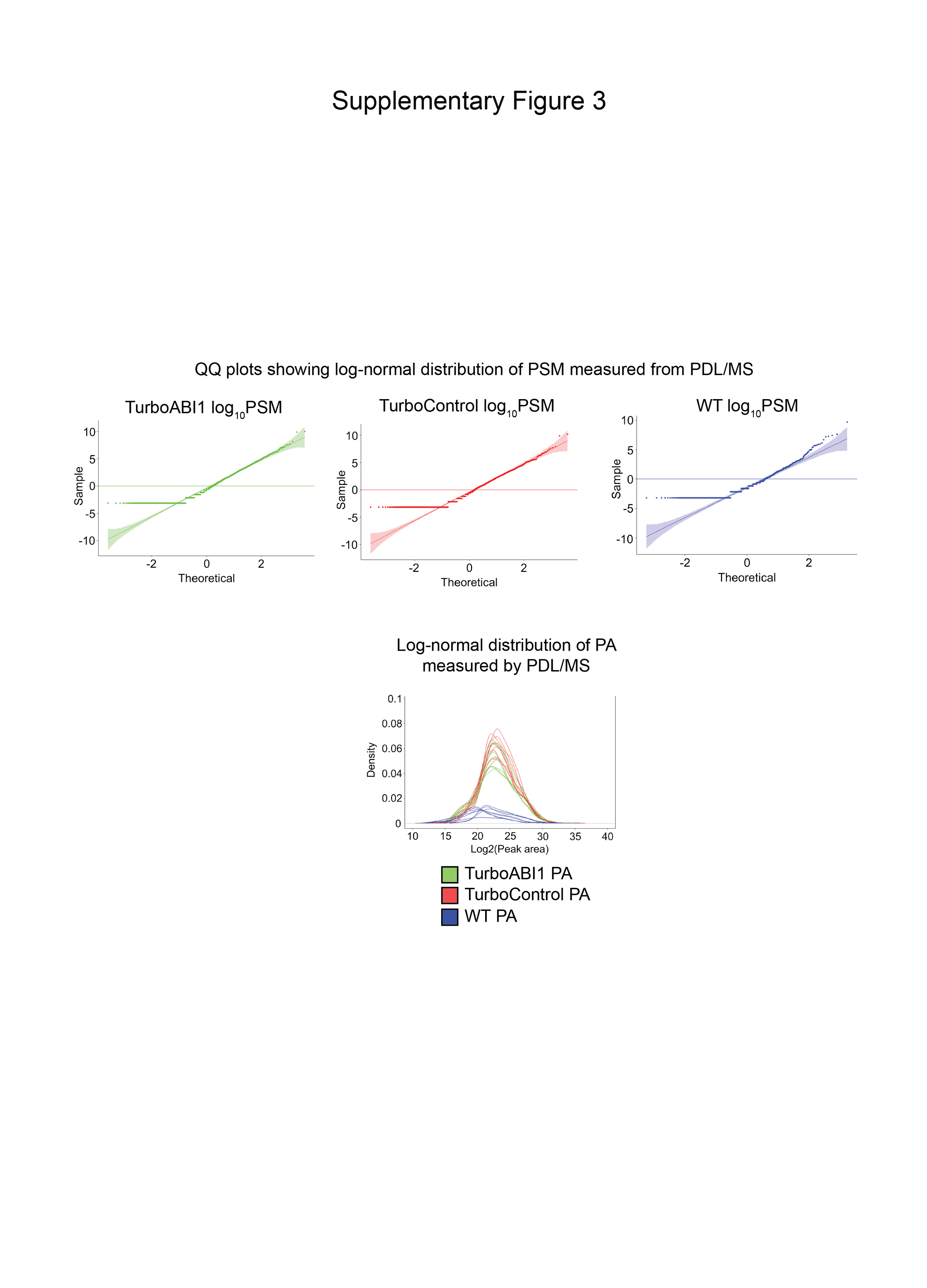

Supplement: Supplementary file 12 — Fig. S3. Lognormality of PSM and PA data generated from labeling and MS of TurboABI1 #1, TurboControl #1, and NIH/3T3 WT cell lines. [file MOL2-17-2356-s012.tif]

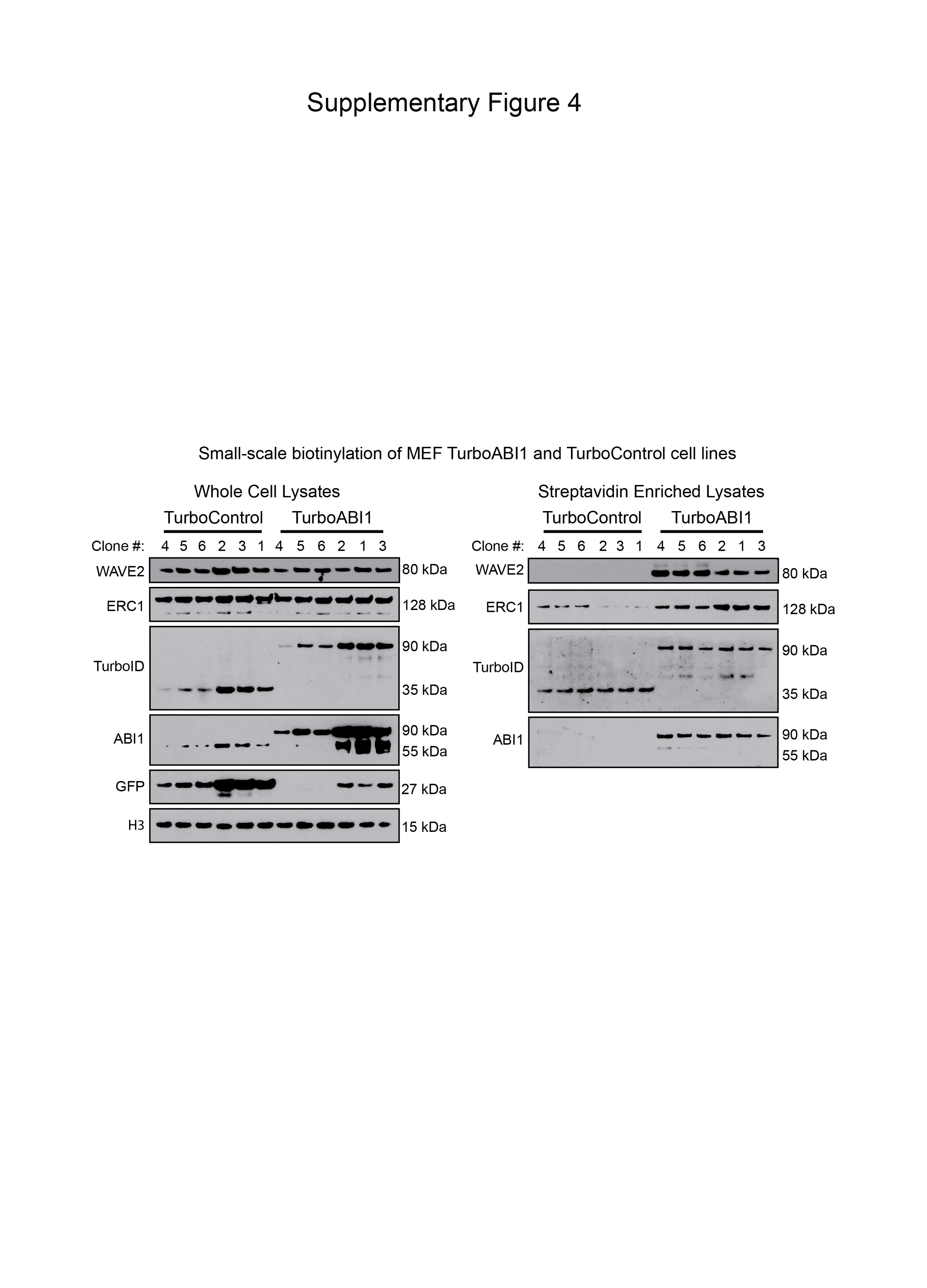

Supplement: Supplementary file 13 — Fig. S4. Small scale biotin labeling of induced TurboControl and TurboABI1 cell lines. [file MOL2-17-2356-s013.tif]

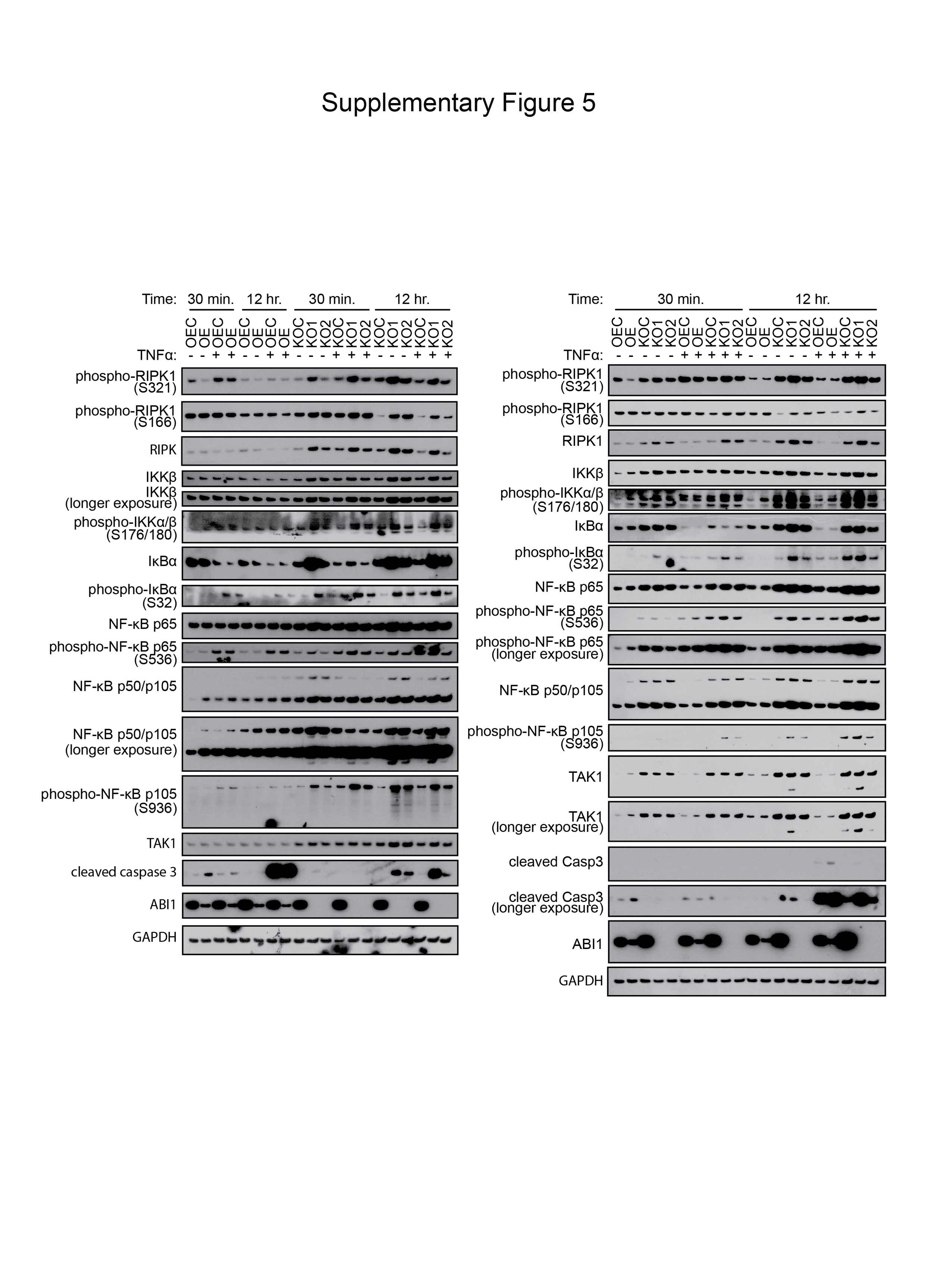

Supplement: Supplementary file 14 — Fig. S5. Western blots of NF‐κB pathway components and activation in ABI1 OE, KO, and control cell lines stimulated with TNFα. [file MOL2-17-2356-s008.tif]

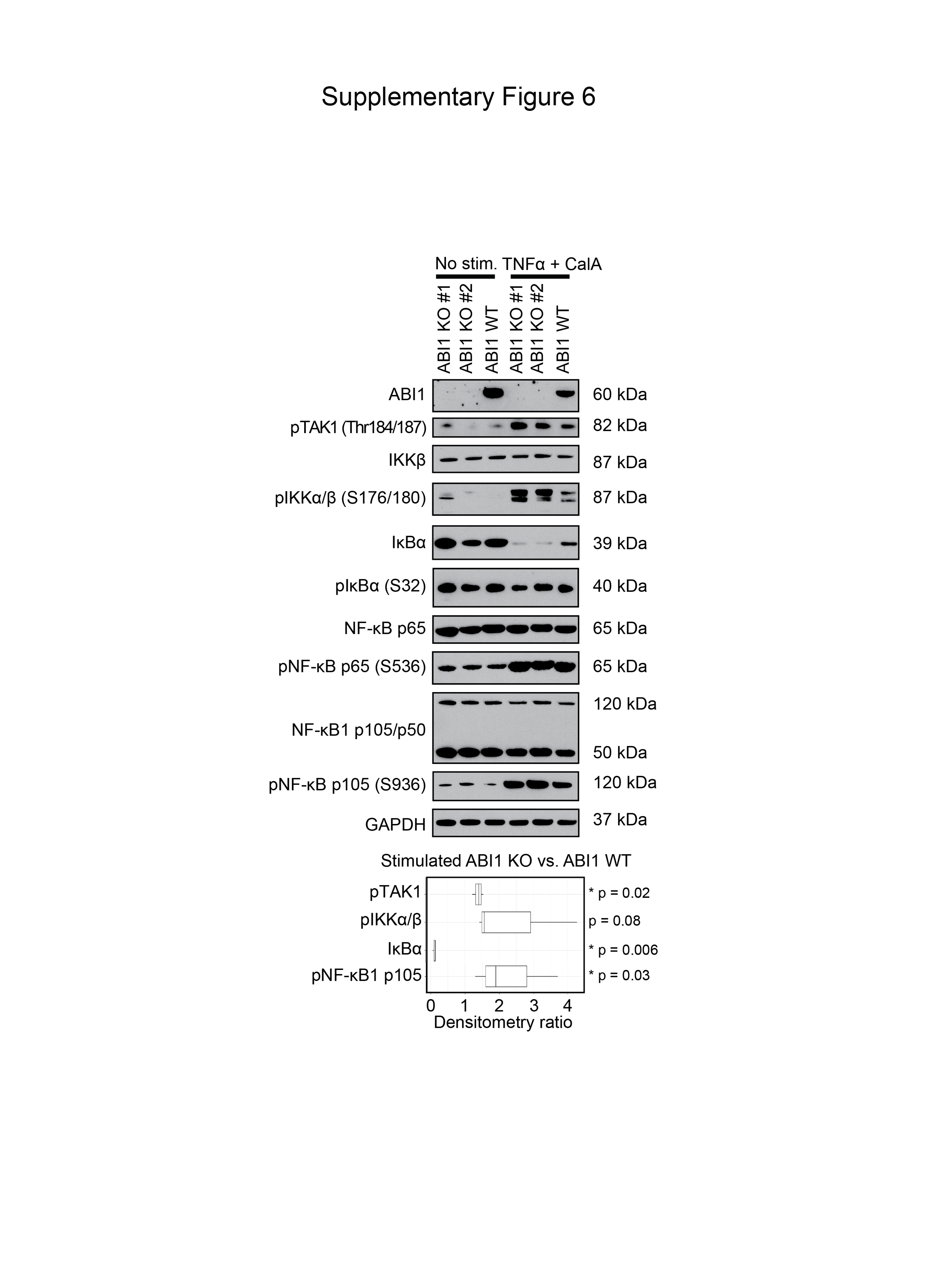

Supplement: Supplementary file 15 — Fig. S6. Western blots of ABI1 KO and WT cell lines stimulated with TNFα and Calyculin A. [file MOL2-17-2356-s005.tif]
